# Educational inequalities in disability at the end of life across European welfare regimes

**DOI:** 10.1016/j.ssmph.2026.101955

**Published:** 2026-07-29

**Authors:** Ewoud T. Jansma, Cecilia Potente, Thijs van den Broek, Anna P. Nieboer

**Affiliations:** Erasmus School of Health Policy & Management, Erasmus University Rotterdam, Burgemeester Oudlaan 50, Rotterdam, 3062 PA, the Netherlands

**Keywords:** Educational inequalities, Disability, Last year of life, End-of-life, Welfare regimes, Europe

## Abstract

Individuals with higher levels of educational attainment experience lower levels of disability than their lower educated counterparts, but it remains unclear how this educational gradient manifests at the end of life. We propose two competing perspectives that offer contrasting expectations. The *cumulative dis/advantage* hypothesis suggests that educational differences in disability continue to widen at the end of life, whereas the *terminal convergence* hypothesis postulates diminishing differences as proximity to death overrides prior social advantages through universal mortality related decline. We test these hypotheses using harmonized end-of-life interviews from 6957 deceased participants in the Survey of Health, Ageing and Retirement in Europe. Additionally, we examine whether educational inequalities vary across welfare regimes. Multilevel beta-binomial models are used to capture individual- and country-level variation in limitations in activities of daily living (ADL) and instrumental activities of daily living (IADL). During the final year of life, medium-educated and high-educated decedents were predicted to have 0.22–0.37 (8–10%) fewer ADL limitations and 0.50–0.63 (17–18%) fewer IADL limitations than low-educated decedents. Conditioning on the disability status at the last observation alive attenuated inequalities, though it did not eliminate them. This suggests that educational inequalities in disability continue to widen in the final phase of life. Welfare regimes moderated these inequalities, with strong evidence for ADL disability and limited evidence for IADL disability. Within-regime differences showed significant educational inequalities in Bismarckian and Eastern European regimes, but not in Southern European or Nordic regimes. Overall, the findings support the *cumulative dis/advantage* hypothesis and highlight the role of institutional context in shaping inequalities in end-of-life disability.

## Highlights

• The educational gradient in disability persists at the end of life in Europe.
• Inequalities widen near death, supporting the cumulative dis/advantage hypothesis.
• Welfare regimes moderate the educational gradient in end-of-life disability.
• Bismarckian and Eastern European regimes show significant educational inequalities.
• Nordic and Southern European regimes show no significant educational inequalities.

## 1 Introduction

Individuals with higher levels of educational attainment can expect to live longer and spend more years free of disability than their lower-educated counterparts (Mäki et al., 2013; Stonkute et al., 2023). Although the educational gradient in disability is well established (Cambois et al., 2016; Rubio Valverde et al., 2021), less is known about how this pattern manifests at the end of life. It is not self-evident that educational inequalities persist in the final stage of life, because proximity to death may come with substantial health deterioration across educational groups. Understanding educational inequalities in end-of-life disability is important, as disability not only impacts health related quality of life (Mar et al., 2010) but also increases the likelihood of initial and recurrent hospitalizations (Porto et al., 2025), which is of particular policy concern considering that healthcare expenditures peak in the last year of life (Polder et al., 2006; Seshamani & Gray, 2004).

Moreover, as explained in further detail later, welfare state arrangements may shape the extent to which educational inequalities translate into differences in later-life health outcomes (e.g., Sieber et al., 2020). Yet, empirical evidence on how institutional contexts shape and potentially mitigate educational inequalities in disability at the end of life remains limited. Prior studies covering educational differences in end-of-life disability have relied exclusively on single country designs and produced mixed results for different contexts. Some studies report a persistent gradient, with lower-educated individuals experiencing worse disability outcomes at the end of life (Lunney et al., 2003; Raab et al., 2018; Smith et al., 2013; Stolz et al., 2025; Wolf et al., 2015), while others find minimal differences (Vierboom, 2022), no significant difference (Liu et al., 2018), or even an opposite pattern (Saito et al., 2022). Substantial methodological heterogeneity precludes comparisons across these studies, leaving it unclear whether divergent results reflect genuine contextual differences or methodological artifacts. A comparative, cross-national design is therefore necessary; only by examining multiple countries within a harmonized framework can it be evaluated whether educational inequalities manifest consistently or vary across institutional contexts.

The current study provides the first cross-national comparison and develops an analytical framework that integrates individual life-course processes with cross-national institutional variation. At the individual level, we draw on competing theoretical perspectives to derive contrasting expectations about whether educational inequalities in disability widen or diminish as death approaches. At the contextual level, we examine whether these processes are conditioned by institutional arrangements, specifically by welfare regimes. Using harmonized end-of-life interviews from 6957 deceased participants in the Survey of Health, Ageing and Retirement in Europe (SHARE) – the only large-scale cross-national survey with harmonized end-of-life interviews – we examine how the educational gradient in disability manifests at the end of life and whether welfare regimes moderate these inequalities.

## 2 Theoretical framework

### 2.1 The educational gradient in end-of-life disability

Two competing theoretical perspectives lead to contrasting expectations about the educational gradient in end-of-life disability. The *cumulative dis/advantage* hypothesis posits that educational inequalities continue to widen at the end of life, reflecting the accumulation of advantages and disadvantages across the full life-course. In contrast, we formulate an alternative and competing *terminal convergence* hypothesis, which proposes that the educational gradient will diminish at the end of life, as proximity to death overrides prior social differences through universal mortality-related decline.

The conceptual anchor for the cumulative dis/advantage hypothesis is the disablement process (Verbrugge & Jette, 1994). This model conceptualizes disability as a sequential pathway (from pathology, to impairments, functional limitations, and ultimately disability), while identifying risk factors (individual characteristics increasing the likelihood or severity of disability), interventions (buffers against disability), and exacerbators (barriers that accelerate or sustain disability) that modify transition probabilities. Although educational attainment is typically completed in early adulthood and remains largely stable thereafter, it plausibly functions as a distal risk factor shaping disablement risks through multiple pathways across the life-course. Education can indirectly provide persistent skills to detect and address health problems (Cambois et al., 2020). It furthermore shapes socioeconomic position, work conditions, and exposure to related occupational and lifestyle hazards (Cambois et al., 2020). Education also facilitates access to interventions, including assistive equipment (e.g., canes, walkers, grab bars), which have been associated with lower reported disability (Agree, 1999; Verbrugge et al., 1997).

Feedback loops in the disablement process serve as the proximate mechanism for cumulative divergence as theorized by Dannefer (2003). An initial impairment or disability can worsen existing limitations or trigger new disablement pathways (Verbrugge & Jette, 1994), producing accumulation over time. Educational attainment may thus not only differentially shape the likelihood of initial disablement but also the subsequent accumulation of disability across the life-course. This would result in widening inequalities in disability between educational groups as people age. Applied to the end-of-life context, the cumulative dis/advantage hypothesis posits that educational inequalities in disability continue to widen at the end of life, and that higher-educated individuals have a lower level of disability than their lower-educated counterparts prior to death.

Although cumulative dis/advantage provides a strong framework for how the educational gradient in disability may widen over the life-course, its explanatory power may diminish at the end of life, where proximity to death, rather than chronological age, better captures functional decline (Wolf et al., 2015). The end-of-life period is characterized by terminal decline, a mortality-related deterioration that typically begins several years prior to death and manifests across multiple domains, including cognitive functioning, emotional well-being, life satisfaction, weight loss, and physical functioning (Cohen-Mansfield et al., 2018). If such pre-death patterns are largely universal, they may reduce the impact of earlier social advantages.

A small body of empirical work supports this notion. For example, Lunney et al. (2018) found that racial disparities in mobility disability in United States disappeared among decedents in the final 18 months of life, while disparities persisted among survivors, suggesting that this convergence reflects proximity to death rather than chronological aging or baseline disability differences. Similarly, Stolz et al. (2025) observed in Austria that, although educational inequalities in the duration of disability – measured via administrative long-term care records – persisted in the final years of life, the magnitude of this gradient weakened as death approached. Together, these findings suggest that mortality-related decline may attenuate prior social stratification at the end of life. Building on this reasoning, the terminal convergence hypothesis postulates diminishing educational inequalities in disability at the end of life, and minimal differences in the level of disability between lower and higher educated decedents.

### 2.2 The role of welfare regimes

Prior empirical evidence of cross-national variation in the strength of the association between education and end-of-life frailty (Jenkins et al., 2023), which is closely linked to disability (Makizako et al., 2015), suggests that the broader institutional context may shape how educational inequalities in disability manifest at the end of life. Welfare regimes – institutional arrangements through which welfare production is allocated between state, market, and families (Esping-Andersen, 1999) – may play an important role, as they partly shape how socioeconomic resources translate into health outcomes in older age (e.g., Sieber et al., 2020).

If cumulative dis/advantage processes continue to operate until death, welfare regimes may influence the magnitude of educational inequalities in end-of-life disability through their moderation of the socioeconomic pathway described above. Welfare regimes that provide higher levels of decommodification — that is, the degree to which social entitlements (Esping-Andersen, 1999) and healthcare access (Bambra, 2005) are provided independent of labor market participation — may reduce dependence on individual socioeconomic resources for standard of living and healthcare access, thereby attenuating educational inequalities in disability at the end of life. Conversely, in welfare regimes with lower levels of decommodification, disability outcomes may be more contingent on individual resources, potentially strengthening the educational gradient. Although decommodification operates primarily during working life, its effects are cumulative insofar as it structures the accumulation of resources and exposures that shape disablement trajectories across the full life-course. Accordingly**,** welfare regime differences in the extent to which socioeconomic disadvantage is buffered may therefore be expected to shape inequalities in the prevalence and progression of disability at the end of life.

These expectations can be mapped onto specific European welfare regimes. Nordic regimes (e.g., Denmark, Sweden), given their universalistic benefits, high decommodification, egalitarianism, and comprehensive social protection (Esping-Andersen, 1999), are expected to display the smallest educational gradients in disability at the end of life. By contrast, post-communist Eastern European regimes (e.g., Hungary, Poland) have experienced major economic upheaval and remain characterized by hybrid and rapidly changing welfare systems, with post-1990 reforms emphasizing marketization, decentralization, and changes to health insurance, tending toward more limited health service provision and less comprehensive social protection (Eikemo et al., 2008; Mackenbach, 2019). These conditions are expected to produce a more pronounced educational gradient in disability at the end of life. Bismarckian regimes (e.g., France, Germany), with earnings-related, employer-administered social insurance schemes, limited redistribution, and an emphasis on the role of the family, and Southern European regimes (e.g., Italy, Spain), characterized by fragmented and internally polarized welfare provision, partial coverage, strong reliance on the family, and clientelism in selective benefit distribution (Eikemo et al., 2008; Ferrera, 1996), are expected to show moderate educational inequalities. If, instead, mortality-related decline becomes the predominant force, institutional differences may only play a small role, and inequalities would show little variation across welfare regimes at the end of life.

## 3 Data and methods

### 3.1 Data and sample

This study uses data from SHARE, a cross-national and longitudinal survey of individuals aged 50 and older (Bergmann et al., 2019; Børsch-Supan et al., 2013). SHARE provides harmonized micro-data on health, socioeconomic status, and social and family networks, covering 28 European countries and Israel. Since its launch in 2004, SHARE has conducted nine waves of data collection using computer-assisted personal interviews, supplemented with self-completion questionnaires, and proxy end-of-life interviews conducted in person or by phone with relatives or close contacts after a respondent's death.

The end-of-life interviews from Waves 8 and 9 (2018–2022) of SHARE serve as the primary data source for our analysis (*N* = 7666). These two waves were selected because they cover the largest number of participating European countries (*N* = 27), thereby facilitating the most comprehensive cross-national analysis. Wave 7 added eight new countries, but end-of-life interviews had not yet been conducted for these countries in that wave. Data from all other waves were used to supplement pre-death information on decedents. European decedents were included in the analysis if they had valid end-of-life interviews in Waves 8 or 9, along with corresponding complete and valid pre-death information from any available wave. Decedents with missing or invalid information on variables of interest were deleted listwise (n = 334; 4.58%). The final analytical sample comprises 6957 decedents across 27 European countries (a detailed flowchart of the sample selection process is provided in Supplementary Fig. S1).

### 3.2 Measures

End-of-life disability was assessed using limitations in ADL and IADL, two widely used indicators of disability in older populations (Yokota & Van Oyen, 2020). ADLs capture basic self-care tasks essential for personal independence, including dressing, walking across a room, bathing or showering, eating, getting in and out of bed, and using the toilet. IADLs reflect more complex activities required for independent living. The core set of IADL activities consistently measured across all SHARE waves consists of preparing a hot meal, grocery shopping, making telephone calls, taking medication, using a map, performing household or garden work, and managing money. Proxies for the deceased respondents reported whether the individual experienced any difficulties with these activities due to physical, mental, emotional, or memory problems during the last twelve months of life, with limitations required to have lasted at least three months. For each decedent, the reported number of ADL and IADL limitations was summed, ranging from 0 (no limitations) to 6 for ADLs and 0 to 7 for IADLs, with higher counts indicating greater disability.

The disability status at the last observation alive was operationalized as the number of limitations reported in the final SHARE wave in which the respondent participated while alive. To account for variation in timing, the observation gap – the number of years between the last interview assessing disability and the respondent's date of death – was included. Incorporating these measures in selected models allows for distinguishing educational differences in disability progression at the end of life from educational differences in previously accumulated disability, which would otherwise be conflated in the estimated educational gradient.

Educational attainment was measured based on the respondent's self-reported highest school-leaving certificate or academic degree. SHARE harmonized these responses using the International Standard Classification of Education (ISCED-97), facilitating cross-national comparisons of educational levels across countries and survey waves. Following the ISCED framework, educational attainment is categorized as low (no schooling, primary, or lower-secondary education; ISCED ≤2), medium (upper-secondary or post-secondary non-tertiary education; ISCED 3–4), and high (tertiary education, including university and advanced research degrees; ISCED 5–6) (UNESCO, 1997).

Additional information on the deceased respondent includes sex (male or female), age at death (in years), marital status (married or not at the time of death), country of residence as indicated by the country identifier (corresponding to the country in which the respondent participated in SHARE), and the relationship of the proxy respondent to the deceased (spouse, other relative, or non-relative).

The 27 countries in the final sample were classified into four welfare regimes, following an extended version of Eikemo et al. (2008) adaptation of Ferrera's (1996) typology.^1^ Countries were grouped as follows: Eastern European (Bulgaria, Croatia, Czech Republic, Estonia, Hungary, Latvia, Lithuania, Poland, Romania, Slovakia, and Slovenia), Bismarckian (Austria, Belgium, France, Germany, Luxembourg, the Netherlands, and Switzerland), Southern European (Cyprus, Greece, Italy, Malta, Portugal, and Spain), and Nordic (Denmark, Finland, and Sweden).

### 3.3 Statistical analysis

The distributions of ADL and IADL limitations during the last year of life are distinctly U-shaped (see Supplementary Fig. S2), with many decedents having experienced either no limitations or limitations in all activities, and relatively few with intermediate levels of disability. To accommodate these bounded counts and the accumulation at both extremes, beta-binomial models were employed (Liu et al., 2013). In this specification, the observed number of limitations is treated as a binomial outcome on a fixed number of possible activities, while the underlying probability of limitation for each individual is assumed to follow a beta distribution, allowing the model to flexibly capture overdispersion and U-shaped patterns in the data.

Given that decedents were clustered within countries, multilevel modeling with country-level random intercepts was used to account for the dependence of observations within countries and to properly model the variance in disability outcomes at both the individual and country levels. In addition, interactions between individual educational attainment and country-level welfare regime (included as a fixed effect) were modeled to examine whether educational inequalities in disability differ across regimes.

Formally, the number of limitations $Y_{ij}$ for individual i in country j is modeled as:

$$Y_{ij}∼Betabinomial\left(m,p_{ij},\phi \right)\text{,}$$

where m represents the maximum number of limitations (6 for ADL, 7 for IADL), $p_{ij}$ is the per-activity probability that individual i in country j experiences a limitation, and ϕ is the dispersion parameter capturing overdispersion (Najera-Zuloaga et al., 2017). The probability $p_{ij}$ is linked to covariates via a logit link:

$$\text{logit}\left(p_{ij}\right)=x_{ij}^{\prime }\beta +u_{j}\text{,}$$

where $x_{ij}$ is the vector of covariates, β is the vector of fixed effect coefficients, and $u_{j}∼N\left(0,\sigma_{u}^{2}\right)$ is a country-level random intercept capturing unobserved country-specific deviations.

The analytical approach consists of a sequence of models structured to progressively examine the educational gradient in disability at the end of life and its potential moderation by welfare regimes. The initial models estimate the association between educational attainment and disability during the final year of life adjusted for sex, age at death, marital status at death, and relationship to proxy. Subsequent models also condition on the disability status at the last observation alive, thereby accounting for earlier accumulated differences. This changes the interpretation of the educational gradient. In the first models, it captures predicted differences between educational groups in the *level* of disability in the last year of life. These differences reflect the combination of differences in disability in *progression* in the final phase of life and differences accumulated already prior to this phase. In models that condition on prior disability, the estimates for education only reflect predicted differences in disability progression in the final phase of life, here operationalized as the period between the last observed interview prior to the decedent's death and the last year of life.

All analyses were conducted in *R* (version 4.4.0). Multilevel beta-binomial regression models were fitted using the *glmmTMB* package (Brooks et al., 2017). Post-estimation quantities were derived using the *marginaleffects* package (Arel-Bundock et al., 2024), based on population-average predictions (country-level random intercepts set to zero). For each educational contrast relative to the low-educated reference group, we report absolute and relative differences. Absolute differences are computed as average differences in predicted probabilities on the response scale, multiplied by the number of activities (6 for ADL, 7 for IADL) to yield expected differences in limitation counts. Relative differences are reported as risk ratios, computed as the ratio of average predicted probabilities of limitation on a given ADL or IADL activity for each education group to those of the low-educated reference group. Within-regime estimates are obtained by stratifying both quantities by welfare regime.^2^ Moderation by welfare regime is assessed via likelihood ratio tests, implemented using the *lmtest* package (Zeileis & Hothorn, 2002), comparing nested models with and without educational attainment × welfare regime interaction terms; significant test results indicate that educational gradients differ across regimes.

## 4 Results

### 4.1 Descriptive characteristics of decedents

Table 1 summarizes the descriptive characteristics of the 6957 decedents included in the analytical sample. On average, decedents had 2.52 ADL limitations (SD = 2.53) and 3.39 IADL limitations (SD = 2.90) in the last year of life. A substantial proportion had no ADL limitations (40.9%), compared with 31.6% with no IADL limitations, consistent with the notion that IADL limitations generally precede ADL limitations (Yokota & Van Oyen, 2020). The level of disability in the last observation alive tended to be lower: decedents had, on average, 1.36 ADL limitations (SD = 1.85) and 1.78 IADL limitations (SD = 2.36). The mean observation gap between the last disability assessment and death was 2.50 years (SD = 2.15).

**Table 1:** Descriptive statistics of decedents in SHARE Waves 8 and 9 (*N* = 6957).

Variables	Mean (SD)/N (%)	
*Disability (last year of life)*		
ADL limitations	2.52	(2.53)
IADL limitations	3.39	(2.90)
*Disability (last observation alive)*		
ADL limitations	1.36	(1.85)
IADL limitations	1.78	(2.36)
*Observation gap*	2.50	(2.15)
*Educational attainment*		
Low	3701	(53.2%)
Medium	2328	(33.5%)
High	928	(13.3%)
*Sex*		
Male	3646	(52.4%)
Female	3311	(47.6%)
*Age at death (years)*	80.4	(9.85)
*Marital status*		
Married	3599	(51.7%)
Not married	3358	(48.3%)
*Proxy*		
Spouse	2740	(39.4%)
Other relative	3155	(45.4%)
Non-relative	1062	(15.3%)
*Welfare regimes*		
Eastern	3445	(49.5%)
Bismarckian	1419	(20.4%)
Southern	1416	(20.4%)
Nordic	677	(9.73%)

Educational attainment was skewed toward the lower end: just over half of decedents had low education (53.2%), about a third had medium education (33.5%), and 13.3% had high education. Men comprised 52.4% of the sample, the mean age at death was 80.4 years (SD = 9.85), and slightly more than half of the decedents (51.7%) were married at the time of death. Nearly half of the decedents were from Eastern European regimes (49.5%), while smaller proportions were from Southern European (20.4%), Bismarckian (20.4%), and Nordic (9.7%) regimes.

### 4.2 The educational gradient in disability at the end of life

**Fig. 1:**
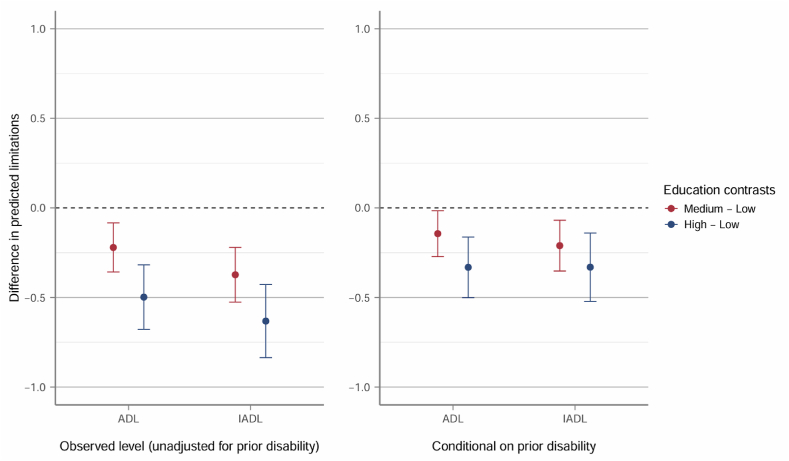
Absolute differences in predicted ADL and IADL limitations by educational attainment relative to the low-educated reference group (*N* = 6957). Notes: All models adjust for sex, age at death, marital status, and relationship to proxy. Models conditional on prior disability include the disability status at the last observation alive and observation gap as additional covariates. Error bars represent 95% confidence intervals. Data are from the Survey of Health, Ageing and Retirement in Europe.

Fig. 1 presents the absolute differences in predicted ADL and IADL limitations by educational attainment (see Supplementary Table S1 for detailed regression results; Supplementary Figs. S3 and S4 for relative differences). The results reveal a clear gradient: higher educational attainment was significantly associated with fewer ADL and IADL limitations during the final year of life. For ADL, medium-educated decedents were predicted to have 0.221 fewer limitations on average compared with low-educated decedents (95% CI [−0.358, −0.084]), while high-educated decedents had 0.498 fewer limitations during the last year of life (95% CI [−0.678, −0.318]). A similar gradient was observed for IADL, with medium-educated decedents exhibiting 0.373 fewer limitations (95% CI [−0.526, −0.220]) and high-educated decedents 0.632 fewer limitations (95% CI [−0.836, −0.427]) compared to low-educated decedents. The relative gradient was broadly comparable across outcomes: medium-educated decedents had approximately 8% and 10% fewer predicted limitations during the final year of life relative to low-educated decedents for ADL and IADL, respectively (ADL: RR = 0.92, 95% CI [0.87, 0.97]; IADL: RR = 0.90, 95% CI [0.86, 0.94]), while the corresponding figures were approximately 18% and 17% for high-educated decedents (ADL: RR = 0.82, 95% CI [0.75, 0.88]; IADL: RR = 0.83, 95% CI [0.77, 0.88]).

After conditioning on the disability status at the last observation alive, thereby accounting for educational differences in disability accumulated prior to the terminal phase, educational inequalities remained statistically significant but became smaller in magnitude. For ADL, predicted limitations were on average 0.144 lower for medium-educated decedents (95% CI [−0.272, −0.016]) and 0.332 lower for high-educated decedents (95% CI [−0.501, −0.162]) compared with low-educated decedents. A comparable pattern was observed for IADL, with medium- and high-educated decedents predicted to have on average 0.211 and 0.331 fewer limitations relative to low-educated decedents at the end of life, respectively (medium: 95% CI [−0.352, −0.069]; high: 95% CI [−0.522, −0.140]). The relative gradient was similarly attenuated: medium-educated decedents had approximately 5–6% fewer predicted limitations than low-educated decedents across both outcomes (ADL: RR = 0.95, 95% CI [0.90, 0.99]; IADL: RR = 0.94, 95% CI [0.90, 0.98]), with corresponding relative differences of 13% for ADL and 9% for IADL among high-educated decedents (ADL: RR = 0.87, 95% CI [0.81, 0.94]; IADL: RR = 0.91, 95% CI [0.85, 0.96]).

Taken together, these findings reveal a substantial educational gradient in disability at the end of life, with higher educated decedents consistently exhibiting fewer limitations across both outcomes. When models additionally account for disability status at the last observation alive, the gradient attenuates but remains statistically significant. This suggests that mortality-related decline does not override prior differences at the end of life, providing evidence against the terminal convergence hypothesis. Instead, educational inequalities in disability appear to continue widening at the end of life, supporting the cumulative dis/advantage hypothesis.

### 4.3 Moderation by welfare regime

To examine whether the educational gradient in end-of-life disability varies across welfare regimes, interactions between individual educational attainment and country-level welfare regime were estimated (see Supplementary Tables S2 and S3 for detailed regression results). Likelihood ratio tests indicated that, for ADL, inclusion of the interaction terms significantly improved model fit (χ^2^(6) = 13.83, p < 0.05). This provides statistical evidence that the association between educational attainment and ADL disability during the final year of life differs across welfare regimes. For IADL, the interaction did not significantly improve model fit (χ^2^(6) = 11.01, p = 0.09). After conditioning on the disability status at the last observation alive, the interaction remained significant for ADL disability (χ^2^(6) = 13.06, p < 0.05), whereas no significant interaction was observed for IADL disability (χ^2^(6) = 8.16, p = 0.23). This indicates that welfare regimes moderate the educational gradient in ADL disability at the end of life, even net of educational differences in disability accumulated across the life-course, whereas no evidence of such moderation was found for IADL disability.

**Fig. 2:**
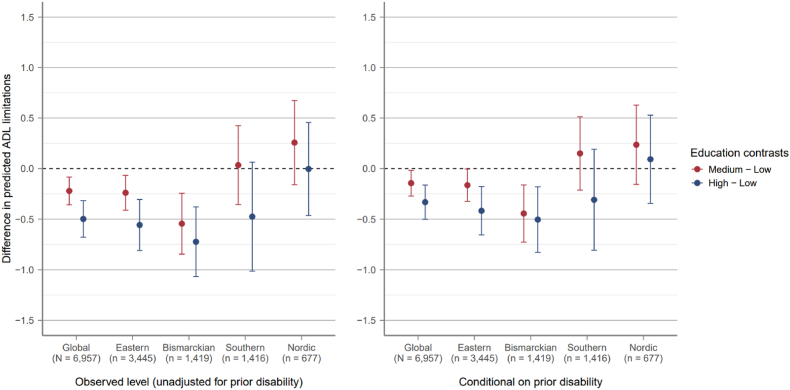
Absolute differences in predicted ADL limitations by educational attainment relative to the low-educated reference group, by European welfare regime. Notes: All models adjust for sex, age at death, marital status, and proxy relationship. Models conditional on prior disability additionally adjust for disability status at the last observation alive and observation gap. Error bars represent 95% confidence intervals. Data are from the Survey of Health, Ageing and Retirement in Europe.

**Fig. 3:**
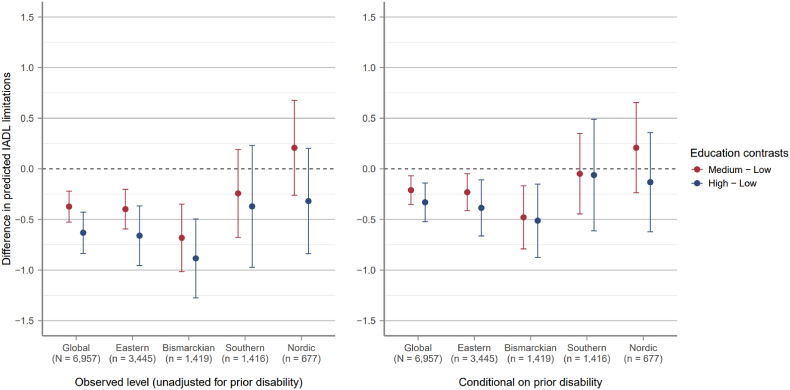
Absolute differences in predicted IADL limitations by educational attainment relative to the low-educated reference group, by European welfare regime. Notes: All models adjust for sex, age at death, marital status, and proxy relationship. Models conditional on prior disability additionally adjust for disability status at the last observation alive and observation gap. Error bars represent 95% confidence intervals. Data are from the Survey of Health, Ageing and Retirement in Europe.

Fig. 2, Fig. 3 present within-regime differences in predicted ADL and IADL limitations by educational attainment, respectively. Models not adjusted for prior disability revealed clear educational gradients in both ADL and IADL disability during the final year of life within Bismarckian and Eastern European regimes. No statistically significant educational inequalities were observed within Southern European or Nordic regimes.

Fig. 2 shows the results for ADL. Medium-educated decedents in Bismarckian regimes were predicted to have 0.545 fewer ADL limitations on average compared with low-educated decedents (95% CI [−0.846, −0.244]), while high-educated decedents had 0.723 fewer predicted ADL limitations during the last year of life (95% CI [−1.068, −0.378]). Corresponding differences were somewhat smaller in Eastern European regimes, where medium- and high-educated decedents were predicted to have 0.239 and 0.558 fewer ADL limitations relative to low-educated decedents, respectively (medium: 95% CI [−0.411, −0.067]; high: 95% CI [−0.810, −0.305]). Risk ratios indicated that medium-educated decedents in Bismarckian regimes had on average 16% fewer predicted ADL limitations compared with low-educated decedents (RR = 0.84, 95% CI [0.75, 0.92]), with the corresponding estimate being 22% for high-educated decedents (RR = 0.78, 95% CI [0.69, 0.88]). In Eastern European regimes, comparable relative gradients were observed, with medium-educated decedents having 10% fewer predicted ADL limitations (RR = 0.90, 95% CI [0.83, 0.97]) and high-educated decedents 24% fewer (RR = 0.76, 95% CI [0.66, 0.87]) relative to low-educated decedents.

Fig. 3 shows a largely similar pattern for IADL disability. In Bismarckian regimes, medium-educated decedents were predicted to have 0.682 fewer IADL limitations relative to low-educated decedents during the final year of life (95% CI [−1.016, −0.349]), while high-educated decedents had 0.885 fewer IADL limitations (95% CI [−1.275, −0.495]). In Eastern European regimes, predicted differences were descriptively smaller, with medium-educated decedents exhibiting 0.399 fewer IADL limitations (95% CI [−0.595, −0.202]) and high-educated decedents 0.661 fewer IADL limitations relative to low-educated decedents (95% CI [−0.955, −0.366]). The corresponding relative gradients revealed the same pattern. In Bismarckian regimes, medium-educated decedents had 16% fewer predicted IADL limitations compared with low-educated decedents (RR = 0.84, 95% CI [0.76, 0.91]), while high-educated decedents had 21% fewer (RR = 0.79, 95% CI [0.70, 0.88]). In Eastern European regimes, medium-educated decedents had 12% fewer predicted IADL limitations (RR = 0.88, 95% CI [0.82, 0.94]) and high-educated decedents 20% fewer (RR = 0.80, 95% CI [0.71, 0.89]) relative to low-educated decedents.

Conditioning on the disability status at the last observation alive attenuated estimates in both Bismarckian and Eastern European regimes. No statistically significant educational gradients were observed in Southern European or Nordic regimes. For ADL in the Bismarckian regime, medium-educated decedents were predicted to have 0.444 fewer limitations in the final phase of life relative to low-educated decedents (95% CI [−0.727, −0.161]), while high-educated decedents had 0.505 fewer limitations (95% CI [−0.828, −0.181]). In Eastern European regimes, medium- and high-educated decedents were predicted to have 0.164 and 0.417 fewer ADL limitations relative to low-educated decedents, respectively (medium: 95% CI [−0.324, −0.004]; high: 95% CI [−0.656, −0.179]). The relative gradients mirrored these patterns. Within Bismarckian regimes, medium-educated decedents had on average 13% fewer predicted ADL limitations in the final phase of life compared with low-educated decedents (RR = 0.87, 95% CI [0.78, 0.95]), with the corresponding estimate being 15% for high-educated decedents (RR = 0.85, 95% CI [0.75, 0.94]). In Eastern European regimes, medium- and high-educated decedents had 7% and 18% fewer predicted ADL limitations, respectively (medium: RR = 0.93, 95% CI [0.86, 1.00]; high: RR = 0.82, 95% CI [0.72, 0.92]).

The estimates were similarly attenuated for IADL after conditioning on prior disability but remained statistically significant in both regimes. In the Bismarckian regime, medium-educated decedents were predicted to have 0.479 fewer IADL limitations relative to low-educated decedents (95% CI [−0.791, −0.168]), while high-educated decedents had 0.513 fewer IADL limitations (95% CI [−0.875, −0.151]). In Eastern European regimes, medium- and high-educated decedents were predicted to have 0.232 and 0.386 fewer IADL limitations relative to low-educated decedents, respectively (medium: 95% CI [−0.414, −0.050]; high: 95% CI [−0.663, −0.110]). No statistically significant differences were observed in Southern European or Nordic regimes. The relative gradients repeated these patterns. In the Bismarckian regime, medium-educated decedents had 12% fewer predicted IADL limitations in the final phase of life compared with low-educated decedents (RR = 0.88, 95% CI [0.81, 0.95]), while high-educated decedents had 13% fewer (RR = 0.87, 95% CI [0.78, 0.96]). In Eastern European regimes, medium- and high-educated decedents had 7% and 12% fewer predicted IADL limitations relative to low-educated decedents, respectively (medium: RR = 0.93, 95% CI [0.87, 0.98]; high: RR = 0.88, 95% CI [0.80, 0.96]).

Whereas the largest educational inequalities were expected in Eastern European regimes, followed by intermediate inequalities in Bismarckian and Southern European regimes and the smallest inequalities in Nordic regimes, the empirical results descriptively indicate the most pronounced educational gradients in Bismarckian regimes, somewhat smaller gradients in Eastern European regimes, and no statistically significant educational inequalities in Southern European or Nordic regimes. These patterns were observed both before and after conditioning on the disability status at the last observation alive, although this conditioning attenuated the estimates. Taken together, we can conclude that processes of cumulative dis/advantage continue to operate at the end of life in both Bismarckian and Eastern European regimes, with high-educated decedents displaying substantially fewer ADL and IADL limitations.

While regime-specific estimates tentatively suggest variation in the magnitude of educational gradients, formal interaction tests provide the basis for conclusions about moderation. Likelihood ratio tests supported moderation by welfare regimes for ADL disability at the end of life, but the corresponding interaction for IADL disability was not statistically significant. Hence, statistical evidence for welfare regime as a moderator of educational inequalities is limited to ADL disability, with insufficient evidence to make conclusive statements about moderation for IADL disability.

### 4.4 Robustness checks

Four sets of sensitivity analyses were conducted to assess the robustness of the observed educational gradient in end-of-life disability (see Supplementary Sections 7–11 for corresponding figures and tables). First, because some educational attainment × welfare regime combinations had small cell counts, medium- and high-education groups were collapsed to ensure stable estimates. Re-estimating models with this collapsed education variable yielded results consistent with the main analyses: the medium-to high-educated were predicted to have significantly fewer ADL and IADL limitations compared to low-educated decedents. Within-regime differences substantively mirrored the main analyses. Specifically, that educational inequalities were statistically significant in Eastern European and Bismarckian regimes, both before and after conditioning on prior disability, whereas no significant inequalities were observed in Southern European or Nordic regimes.

Across alternative specifications, likelihood ratio tests consistently indicated statistically significant moderation by welfare regime of educational inequalities in ADL disability. For IADL disability, moderation by welfare regime was established before conditioning on prior disability (χ^2^(3) = 9.48, p < 0.05); however, after accounting for prior disability, the evidence did not reach conventional statistical significance (χ^2^(3) = 6.93, p = 0.07). This suggests that welfare regimes moderate educational inequalities in IADL disability observed in the final year of life between low-educated and medium-to high-educated decedents; however, it remains uncertain whether welfare regimes also moderate educational differences in IADL limitations that emerge specifically at the end of life, as opposed to merely reflecting prior accumulated differences in disability.

Second, given the possibility that the educational gradient in end-of-life disability may vary between men and women or by age-at-death groups, we examined whether the educational gradient in end-of-life disability differed by sex or age at death using interaction models. For the latter, age at death was stratified into three groups (under 75, 75–85, and over 85 years). No statistically significant interactions by age group or by sex of the decedent were observed for either ADL or IADL outcomes.

Third, potential gendered biases in IADL measurement were considered (Sheehan & Tucker-Drob, 2019). Given that certain activities (e.g., preparing a hot meal) have traditionally been more commonly performed by women, particularly in the older cohorts represented in this study, reported difficulty may reflect both gendered role expectations and functional limitations. Models were therefore re-estimated using only the activities on taking medication and making telephone calls, thereby excluding the other, more gendered activities. Results from these restricted models were consistent with the full IADL specification, indicating that the educational gradient persists when potential gender-related measurement biases are minimized.

Fourth, we assessed the sensitivity of the welfare regime interactions to cross-country heterogeneity within welfare regimes. We sequentially re-estimated all models excluding each country in turn and compared regime-specific contrasts in predicted absolute differences to the full-sample estimates. Overall, results were highly robust to the exclusion of individual countries, indicating that no single country exerts disproportionate leverage on the estimated educational gradients in end-of-life disability. Absolute deviations relative to the full-sample estimates were generally small and clustered near zero, typically below 0.02. The largest and most consistent variation was observed in the Nordic regime, where excluding Sweden shifted the estimated contrast upward by between approximately 0.014 and 0.08 across outcomes. This sensitivity reflects movement around a near-zero baseline rather than substantively meaningful changes in the regime effect. In a small number of cases, point estimates crossed zero when baseline estimates were close to zero (e.g., 0.015 to −0.014), but these changes did not alter the overall pattern of regime differences or the substantive conclusions.

## 5 Discussion

This study examined how the educational gradient in disability manifests at the end of life and whether welfare regimes moderate these inequalities. Using harmonized SHARE data from 27 European countries and multilevel beta-binomial models, our analyses captured the full distribution of ADL and IADL limitations and incorporated cross-national variation in institutional contexts. Overall, the results revealed a clear educational gradient: higher educational attainment was consistently associated with fewer ADL and IADL limitations at the end of life. Importantly, these differences persisted after accounting for prior disability, indicating that differences in ADL and IADL disability continue to widen between educational groups, even in the final phase of life. Within-regime differences suggest some variation in the magnitude of this gradient across welfare regimes, with formal interaction tests providing statistical support for moderation by welfare regimes in ADL disability, whereas the evidence provided here does not allow us to be conclusive about moderation in IADL disability.

The overall results are consistent with the cumulative dis/advantage hypothesis. Compared with low-educated decedents, medium-educated and, especially, high-educated decedents were predicted to experience fewer ADL and IADL limitations during the final year of life. Accounting for the disability status at the last observation alive attenuated these differences, but the educational gradient remained statistically significant. This suggests that educational inequalities in disability continue to widen even at the end of life, aligning with the synthesis of the disablement process and cumulative dis/advantage theory (Cambois et al., 2020; Dannefer, 2003; Verbrugge & Jette, 1994). Mortality-related deterioration does not override inequalities accumulated over the life-course; the protective benefits of higher educational groups not only persist but intensify even shortly before death, though their magnitude varies across welfare regimes.

A key contribution of the current study lies in its cross-national comparative design, which enables systematic assessment of how institutional contexts shape educational inequalities in disability at the end of life. Prior single-country studies have yielded conflicting findings, but methodological heterogeneity has precluded determining whether these reflect genuine contextual differences or measurement artifacts. By providing the first harmonized cross-national comparison, this study demonstrates that educational inequalities in end-of-life disability vary systematically across welfare regimes, establishing that institutional arrangements play a substantive role in shaping these inequalities.

Analyses of the interaction between educational attainment and welfare regime revealed heterogeneity in the educational gradient at the end of life across Europe. Within-regime differences indicated that the educational gradient was statistically significant in Bismarckian and Eastern European regimes, whereas Southern European and Nordic regimes exhibited no significant differences. Likelihood ratio tests provided consistent statistical support for welfare-regime moderation of the educational gradient in ADL disability. For IADL disability, however, we cannot be conclusive about whether welfare regimes also moderate the extent to which differences in IADL disability between low-educated and medium-to high-educated decedents continue to widen in the last phase of life.

Reflecting on the findings in light of theoretical expectations concerning welfare regimes, the smallest educational gradient was anticipated in Nordic regimes and the largest in Eastern European regimes, with Bismarckian and Southern European regimes expected to occupy intermediate positions. Within-regime differences did not fully align with these expectations. Point estimates suggest the most pronounced educational gradient in Bismarckian regimes. Possibly, this reflects the influence of earnings-related, employer-administered social insurance schemes that tie access to resources and care to prior labor-market position, thereby amplifying the advantages of higher-educated individuals. The relatively strong gradient in Eastern European regimes aligns with expectations given the limited and uneven provision of social and health services in post-communist regimes, which allows individual resources to play a larger role in shaping disability outcomes. Consistent with expectations, Nordic regimes showed no significant gradient, likely reflecting the buffering effect of generous, universalistic social protection. This indicates that such contexts may mitigate inequalities in disability in the final stages of life. Southern European regimes also exhibited no significant educational inequalities in end-of-life disability. This discrepancy between expectation and result may reflect delayed economic, social, and cultural modernization. Among older cohorts, low-educated individuals remain numerous and less socially marginalized, education is a weaker predictor of occupational class in Southern Europe than in other European regions, and traditional health-protective patterns – such as the persistence of Mediterranean dietary practices among low-educated groups – may explain the absence of an educational gradient in end-of-life disability, in a manner analogous to their suggested role in explaining small mortality inequalities (Mackenbach, 2019).

Several limitations should be acknowledged. First, the analysis relies primarily on proxy reports of end-of-life disability, which may be subject to recall bias and other reporting errors. Such bias may vary systematically by educational attainment, potentially leading to over- or underestimation of educational differences in end-of-life disability. Moreover, end-of-life interviews were not completed for all deceased participants, and coverage varies across countries (Bergmann et al., 2019). Because the analytical design anchors on the end-of-life interview and collects pre-death information retrospectively, conventional longitudinal attrition does not arise; all included decedents completed an end-of-life interview per se. However, non-completion of end-of-life interviews may itself be selective: because most SHARE countries lack centralized mortality registers, the vital status of some non-respondents remains unknown, and whether non-participation in the proxy end-of-life interview is related to the education level of the deceased cannot be determined from available data, which may introduce selection bias. Second, although a clear educational gradient is observed, the study design does not permit causal inference. The predicted differences should therefore not be interpreted as direct effects of educational attainment itself. Third, although welfare regime typologies provide a useful framework for cross-national comparison, they involve a degree of simplification of cross-national institutional variation, as countries within the same regime category may not conform to that classification across all policy domains. Moreover, regime types are a coarse grouping that does not allow identification of which specific institutional components drive differences in educational inequalities in end-of-life disability. Fourth, liberal welfare regimes (e.g., the United Kingdom), characterized by minimal, means-tested state support, and strong reliance on private welfare provision (Esping-Andersen, 1999), were not included in the end-of-life interviews of SHARE Waves 8 and 9 and could not be supplemented with data from the English Longitudinal Study of Ageing due to non-comparable disability measures (e.g., differing reference periods), leaving a gap in understanding how such institutional contexts shape educational inequalities in end-of-life disability.

Future research could extend this work in several directions. First, in light of the limitations noted above, future studies employing quasi-experimental designs (e.g., exploiting changes in compulsory schooling laws to identify the causal effects of education) are needed to advance understanding of socioeconomic health inequalities at the end of life, a domain in which causal evidence remains scarce. Second, extending analyses to additional institutional contexts, such as liberal welfare regimes or other global settings, could reveal how variation in social protection, market reliance, and cultural norms influence socioeconomic inequalities in end-of-life disability. Third, analyses incorporating finer-grained policy indicators (e.g., provision of assistive equipment), could help identify the institutional features most effective in mitigating inequalities in end-of-life disability.

Regardless of these limitations, this study implies that processes of cumulative dis/advantage continue to operate at the end of life. Higher levels of educational attainment are consistently associated with lower levels of end-of-life disability. At the same time, these inequalities appear to vary across European welfare regimes, suggesting that institutional context may moderate the educational gradient. Overall, these findings suggest that educational attainment continues to structure disability outcomes at the end of life in Europe, with the magnitude of these inequalities shaped by institutional context.

## Ethical statement

This study uses secondary data from the Survey of Health, Ageing and Retirement in Europe (SHARE), which received ethical approval from the Ethics Council of the Max Planck Society. No additional ethical approval was required for this secondary analysis. All data were fully anonymized prior to access, and no informed consent from individual participants was required for this study.

## CRediT authorship contribution statement

**Ewoud T. Jansma:** Conceptualization, Formal analysis, Methodology, Visualization, Writing – original draft. **Cecilia Potente:** Conceptualization, Supervision, Writing – review & editing. **Thijs van den Broek:** Conceptualization, Methodology, Supervision, Writing – review & editing. **Anna P. Nieboer:** Conceptualization, Supervision, Writing – review & editing.

## Declaration of interest statement

We declare no conflicts of interest.

## Data Availability

All study code is fully replicable and available in the following GitHub repository: https://github.com/EwoudJansma/edu-inequalities-disability-eol. This paper uses data from SHARE Waves 1, 2, 3, 4, 5, 6, 7, 8 and 9 (DOIs: 10.6103/SHARE.w1.900, 10.6103/SHARE.w2.900, 10.6103/SHARE.w3.900, 10.6103/SHARE.w4.900, 10.6103/SHARE.w5.900, 10.6103/SHARE.w6.900, 10.6103/SHARE.w6.DBS.100, 10.6103/SHARE.w7.900, 10.6103/SHARE.w8.900, 10.6103/SHARE.w8ca.900, 10.6103/SHARE.w9.900, 10.6103/SHARE.w9ca900, 10.6103/SHARE.HCAP1.100) see Börsch-Supan et al. (2013) for methodological details. The SHARE data collection has been funded by the European Commission, DG RTD through FP5 (QLK6-CT-2001-00360), FP6 (SHARE-I3: RII-CT-2006-062193, COMPARE: CIT5-CT-2005-028857, SHARELIFE: CIT4-CT-2006-028812), FP7 (SHARE-PREP: GA N°211909, SHARE-LEAP: GA N°227822, SHARE M4: GA N°261982, DASISH: GA N°283646) and Horizon 2020 (SHARE-DEV3: GA N°676536, SHARE-COHESION: GA N°870628, SERISS: GA N°654221, SSHOC: GA N°823782, SHARE-COVID19: GA N°101015924) and by DG Employment, Social Affairs & Inclusion through VS 2015/0195, VS 2016/0135, VS 2018/0285, VS 2019/0332, VS 2020/0313, SHARE-EUCOV: GA N°101052589 and EUCOVII: GA N°101102412. Additional funding from the German Federal Ministry of Research, Technology and Space (01UW1301, 01UW1801, 01UW2202), the Max Planck Society for the Advancement of Science, the U.S. National Institute on Aging (U01_AG09740-13S2, P01_AG005842, P01_AG08291, P30_AG12815, R21_AG025169, Y1-AG-4553-01, IAG_BSR06-11, OGHA_04-064, BSR12-04, R01_AG052527-02, R01_AG056329-02, R01_AG063944, HHSN271201300071C, RAG052527A) and from various national funding sources is gratefully acknowledged (see www.share-eric.eu).

## Footnotes

1 This classification has two slight modifications. First, the Eastern European regime includes five additional post-communist countries not covered in Eikemo et al. (2008): Bulgaria, Croatia, Latvia, Lithuania, and Romania. Second, Cyprus and Malta are classified in the Southern European regime in line with Gal (2010), who situates them within an “extended Mediterranean family” of states sharing cultural, familial, and political traits that, alongside moderate social spending and fragmented welfare provision, produce broadly similar welfare outcomes despite differences in specific institutional designs.

2 Average predicted counts of ADL and IADL limitations, derived from the same fitted models as average predicted probabilities on the response scale, scaled by the number of activities (6 for ADL, 7 for IADL) to obtain expected counts, are reported in the Supplementary Material (Fig. S7–S11).
