# Supplementary material for: Educational inequalities in disability at the end of life across European welfare regimes
Source: SSM Popul Health. 2026 Jul 29;35:101955. doi: 10.1016/j.ssmph.2026.101955 (PMC13452393; doi:10.1016/j.ssmph.2026.101955)
Supplement: Supplementary file 1 [file mmc1.docx]

**Supplementary material**

1. **Sample selection process**

**Figure S1.** Sample selection flowchart

2. **Distribution of ADL and IADL counts**

**Figure S2.** Distribution of ADL and IADL counts among decedents in SHARE Waves 8 and 9 (*N* = 6,957)

3. **Main regression models**

**Table S1.** Random intercept beta-binomial (logit) regressions of ADL and IADL limitations at the end of life, SHARE decedents (Waves 8 and 9, *N* = 6,957; 27 countries)

|  | **Model** | | | |
| --- | --- | --- | --- | --- |
| **Predictor** | (1) ADL | (2) ADL | (3) IADL | (4) IADL |
| *Educational attainment* |  |  |  |  |
| Low | ref. | ref. | ref. | ref. |
| Medium | 0.85** (0.78−0.94) | 0.90* (0.81−0.99) | 0.80*** (0.73−0.87) | 0.87** (0.79−0.96) |
| High | 0.70*** (0.61−0.80) | 0.77*** (0.68−0.88) | 0.68*** (0.60−0.77) | 0.80*** (0.71−0.91) |
| *Sex* |  |  |  |  |
| Male | ref. | ref. | ref. | ref. |
| Female | 1.30*** (1.19−1.42) | 1.20*** (1.09−1.31) | 1.29*** (1.18−1.40) | 1.15** (1.05−1.25) |
| *Age at death (years)* | 1.04*** (1.04−1.05) | 1.03*** (1.02−1.03) | 1.05*** (1.05−1.05) | 1.03*** (1.03−1.04) |
| *Marital status* |  |  |  |  |
| Married | ref. | ref. | ref. | ref. |
| Not married | 0.99 (0.88−1.11) | 1.00 (0.89−1.13) | 1.02 (0.91−1.14) | 1.00 (0.89−1.12) |
| *Proxy* |  |  |  |  |
| Spouse | ref. | ref. | ref. | ref. |
| Other relative | 0.83** (0.73−0.94) | 0.80*** (0.71−0.91) | 0.98 (0.87−1.11) | 0.95 (0.84−1.08) |
| Non-relative | 0.53*** (0.45−0.62) | 0.53*** (0.45−0.62) | 0.66*** (0.57−0.77) | 0.69*** (0.59−0.80) |
| *Prior disability* |  |  |  |  |
| No limitations |  | ref. |  | ref. |
| 1 limitation |  | 1.70*** (1.50−1.93) |  | 1.50*** (1.33−1.69) |
| 2 limitations |  | 2.29*** (1.95−2.68) |  | 1.94*** (1.69−2.24) |
| 3 limitations |  | 3.11*** (2.57−3.77) |  | 2.19*** (1.87−2.56) |
| 4 limitations |  | 3.33*** (2.63−4.20) |  | 2.84*** (2.38−3.38) |
| 5 limitations |  | 4.85*** (3.89−6.06) |  | 3.55*** (2.93−4.30) |
| 6 limitations |  | 7.41*** (6.15−8.94) |  | 5.37*** (4.31−6.69) |
| 7 limitations |  |  |  | 8.95*** (7.47−10.7) |
| *Observation gap (years)* |  | 1.06*** (1.04−1.08) |  | 1.07*** (1.05−1.09) |
| *Model parameters* |  |  |  |  |
| Intercept (log-odds) | −3.440*** | −3.017*** | −3.915*** | −3.152*** |
| Dispersion ($\varphi$) | 0.523 | 0.596 | 0.639 | 0.735 |
| Country SD | 0.342 | 0.308 | 0.326 | 0.288 |
| *Model fit* |  |  |  |  |
| Log-likelihood | −11,032.37 | −10,648.46 | −12,243.68 | −11,802.34 |
| AIC | 22,084.75 | 21,330.92 | 24,507.36 | 23,640.67 |
| BIC | 22,153.22 | 21,447.33 | 24,575.83 | 23,763.93 |

**Notes**: Odds ratios (exponentiated coefficients) with 95% CIs in parentheses. Intercept presented as log-odds. Significance levels: * p<0.05, ** p<0.01, *** p<0.001.

4. **Interaction models**

**Table S2.** Random intercept beta-binomial (logit) regressions of ADL limitations at the end of life, including welfare regime, SHARE decedents (Waves 8 and 9, *N* = 6,957; 27 countries)

|  | **Model** | | | |
| --- | --- | --- | --- | --- |
| **Predictor** | (1) ADL | (2) ADL | (3) ADL | (4) ADL |
| *Educational attainment* |  |  |  |  |
| Low | ref. | ref. | ref. | ref. |
| Medium | 0.86** (0.78−0.94) | 0.84** (0.74−0.95) | 0.90* (0.81−0.99) | 0.88* (0.77−1.00) |
| High | 0.70*** (0.61−0.80) | 0.65*** (0.53−0.80) | 0.77*** (0.67−0.88) | 0.71*** (0.58−0.87) |
| *Sex* |  |  |  |  |
| Male | ref. | ref. | ref. | ref. |
| Female | 1.30*** (1.19−1.42) | 1.30*** (1.19−1.42) | 1.20*** (1.09−1.31) | 1.19*** (1.09−1.31) |
| *Age at death (years)* | 1.04*** (1.04−1.05) | 1.04*** (1.04−1.05) | 1.03*** (1.03−1.03) | 1.03*** (1.02−1.03) |
| *Marital status* |  |  |  |  |
| Married | ref. | ref. | ref. | ref. |
| Not married | 0.99 (0.88−1.11) | 0.99 (0.88−1.11) | 1.00 (0.89−1.13) | 1.00 (0.89−1.13) |
| *Proxy* |  |  |  |  |
| Spouse | ref. | ref. | ref. | ref. |
| Other relative | 0.83** (0.73−0.94) | 0.82** (0.73−0.93) | 0.81*** (0.71−0.92) | 0.80*** (0.71−0.91) |
| Non-relative | 0.53*** (0.45−0.62) | 0.53*** (0.45−0.62) | 0.53*** (0.46−0.63) | 0.53*** (0.45−0.62) |
| *Welfare regime* |  |  |  |  |
| Eastern | ref. | ref. | ref. | ref. |
| Bismarckian | 1.58** (1.17−2.13) | 1.73*** (1.25−2.40) | 1.58*** (1.22−2.06) | 1.72*** (1.29−2.31) |
| Southern | 1.29 (0.94−1.76) | 1.23 (0.89−1.71) | 1.27 (0.96−1.68) | 1.20 (0.90−1.61) |
| Nordic | 1.07 (0.71−1.62) | 0.88 (0.56−1.36) | 1.15 (0.80−1.65) | 0.96 (0.65−1.42) |
| *Education × Welfare regime* |  |  |  |  |
| Low × Eastern |  | ref. |  | ref. |
| Medium × Bismarckian |  | 0.82 (0.64−1.04) |  | 0.82 (0.64−1.05) |
| Medium × Southern |  | 1.22 (0.91−1.65) |  | 1.28 (0.95−1.73) |
| Medium × Nordic |  | 1.43* (1.04−1.97) |  | 1.36 (0.99−1.88) |
| High × Bismarckian |  | 0.93 (0.68−1.27) |  | 0.97 (0.70−1.33) |
| High × Southern |  | 1.10 (0.71−1.70) |  | 1.12 (0.72−1.73) |
| High × Nordic |  | 1.53* (1.04−2.26) |  | 1.52* (1.03−2.24) |
| *Prior disability* |  |  |  |  |
| No limitations |  |  | ref. | ref. |
| 1 limitation |  |  | 1.70*** (1.50−1.93) | 1.70*** (1.50−1.93) |
| 2 limitations |  |  | 2.29*** (1.96−2.68) | 2.29*** (1.95−2.68) |
| 3 limitations |  |  | 3.12*** (2.58−3.78) | 3.13*** (2.59−3.80) |
| 4 limitations |  |  | 3.32*** (2.63−4.20) | 3.33*** (2.63−4.20) |
| 5 limitations |  |  | 4.87*** (3.90−6.07) | 4.89*** (3.92−6.11) |
| 6 limitations |  |  | 7.41*** (6.16−8.96) | 7.41*** (6.15−8.94) |
| *Observation gap (years)* |  |  | 1.06*** (1.04−1.08) | 1.06*** (1.04−1.08) |
| *Model parameters* |  |  |  |  |
| Intercept (log-odds) | −3.594*** | −3.593*** | −3.166*** | −3.161*** |
| Dispersion ($\varphi$) | 0.523 | 0.525 | 0.596 | 0.597 |
| Country SD | 0.285 | 0.288 | 0.243 | 0.245 |
| *Model fit* |  |  |  |  |
| Log-likelihood | −11,028.21 | −11,021.29 | −10,643.53 | −10,637.00 |
| AIC | 22,082.41 | 22,080.59 | 21,327.05 | 21,326.00 |
| BIC | 22,171.43 | 22,210.69 | 21,464.00 | 21,504.03 |
| Likelihood ratio test |  | 13.83* |  | 13.06* |

**Notes**: Odds ratios (exponentiated coefficients) with 95% CIs in parentheses. Intercept presented as log-odds. Significance levels: * p<0.05, ** p<0.01, *** p<0.001. Likelihood ratio test compares the additive models (Models 1 and 3) to the interaction models (Models 2 and 4).

**Table S3.** Random intercept beta-binomial (logit) regressions of IADL limitations at the end of life, including welfare regime, SHARE decedents (Waves 8 and 9, *N* = 6,957; 27 countries)

|  | **Model** | | | |
| --- | --- | --- | --- | --- |
| **Predictor** | (1) IADL | (2) IADL | (3) IADL | (4) IADL |
| *Educational attainment* |  |  |  |  |
| Low | ref. | ref. | ref. | ref. |
| Medium | 0.80*** (0.73−0.87) | 0.78*** (0.69−0.88) | 0.87** (0.79−0.95) | 0.86* (0.76−0.97) |
| High | 0.68*** (0.60−0.77) | 0.66*** (0.55−0.80) | 0.80*** (0.70−0.91) | 0.77** (0.64−0.93) |
| *Sex* |  |  |  |  |
| Male | ref. | ref. | ref. | ref. |
| Female | 1.29*** (1.18−1.40) | 1.28*** (1.18−1.40) | 1.15** (1.05−1.25) | 1.14** (1.05−1.25) |
| *Age at death (years)* | 1.05*** (1.04−1.05) | 1.05*** (1.05−1.05) | 1.03*** (1.03−1.04) | 1.03*** (1.03−1.04) |
| *Marital status* |  |  |  |  |
| Married | ref. | ref. | ref. | ref. |
| Not married | 1.02 (0.91−1.14) | 1.02 (0.92−1.14) | 1.00 (0.89−1.12) | 1.00 (0.89−1.12) |
| *Proxy* |  |  |  |  |
| Spouse | ref. | ref. | ref. | ref. |
| Other relative | 0.98 (0.87−1.11) | 0.98 (0.87−1.10) | 0.95 (0.84−1.08) | 0.95 (0.84−1.08) |
| Non-relative | 0.66*** (0.57−0.77) | 0.66*** (0.57−0.76) | 0.69*** (0.59−0.80) | 0.69*** (0.59−0.80) |
| *Welfare regime* |  |  |  |  |
| Eastern | ref. | ref. | ref. | ref. |
| Bismarckian | 1.34 (0.98−1.84) | 1.47* (1.05−2.05) | 1.29 (0.98−1.71) | 1.40* (1.03−1.89) |
| Southern | 1.15 (0.82−1.60) | 1.11 (0.79−1.56) | 1.12 (0.83−1.51) | 1.08 (0.79−1.46) |
| Nordic | 1.16 (0.75−1.79) | 0.98 (0.62−1.54) | 1.28 (0.87−1.88) | 1.12 (0.74−1.69) |
| *Education × Welfare regime* |  |  |  |  |
| Low × Eastern |  | ref. |  | ref. |
| Medium × Bismarckian |  | 0.84 (0.66−1.06) |  | 0.85 (0.67−1.08) |
| Medium × Southern |  | 1.10 (0.83−1.47) |  | 1.13 (0.85−1.51) |
| Medium × Nordic |  | 1.45* (1.07−1.98) |  | 1.34 (0.98−1.82) |
| High × Bismarckian |  | 0.88 (0.65−1.19) |  | 0.93 (0.68−1.25) |
| High × Southern |  | 1.21 (0.80−1.82) |  | 1.25 (0.83−1.88) |
| High × Nordic |  | 1.25 (0.87−1.80) |  | 1.19 (0.83−1.72) |
| *Prior disability* |  |  |  |  |
| No limitations |  |  | ref. | ref. |
| 1 limitation |  |  | 1.50*** (1.33−1.69) | 1.50*** (1.33−1.69) |
| 2 limitations |  |  | 1.94*** (1.69−2.24) | 1.95*** (1.69−2.24) |
| 3 limitations |  |  | 2.19*** (1.87−2.56) | 2.18*** (1.86−2.56) |
| 4 limitations |  |  | 2.84*** (2.38−3.38) | 2.83*** (2.37−3.37) |
| 5 limitations |  |  | 3.55*** (2.93−4.30) | 3.52*** (2.91−4.27) |
| 6 limitations |  |  | 5.37*** (4.31−6.69) | 5.38*** (4.32−6.71) |
| 7 limitations |  |  | 8.96*** (7.47−10.8) | 8.95*** (7.46−10.7) |
| *Observation gap (years)* |  |  | 1.07*** (1.04−1.09) | 1.06*** (1.04−1.09) |
| *Model parameters* |  |  |  |  |
| Intercept (log-odds) | −4.020*** | −4.024*** | −3.249*** | −3.252*** |
| Dispersion (*φ*) | 0.639 | 0.640 | 0.735 | 0.736 |
| Country SD | 0.304 | 0.304 | 0.265 | 0.264 |
| *Model fit* |  |  |  |  |
| Log-likelihood | −12,242.05 | −12,236.55 | −11,800.54 | −11,796.46 |
| AIC | 24,510.11 | 24,511.10 | 23,643.07 | 23,646.91 |
| BIC | 24,599.13 | 24,641.21 | 23,786.87 | 23,831.80 |
| Likelihood ratio test |  | 11.01 |  | 8.16 |

**Notes**: Odds ratios (exponentiated coefficients) with 95% CIs in parentheses. Intercept presented as log-odds. Significance levels: * p<0.05, ** p<0.01, *** p<0.001. Likelihood ratio test compares the additive models (Models 1 and 3) to the interaction models (Models 2 and 4).

5. **Relative educational gradient – Risk ratio’s**

**Figure S3.** Relative differences in predicted ADL limitations between medium- to high-educated decedents relative to low-educated decedents, by European welfare regime

**Notes**: All models adjust for sex, age at death, marital status, and proxy relationship. Models conditional on prior disability additionally adjust for disability status at the last observation alive and observation gap. Error bars represent 95% confidence intervals. Data are from the Survey of Health, Ageing and Retirement in Europe.

**Figure S4.** Relative differences in predicted IADL limitations between medium- to high-educated decedents relative to low-educated decedents, by European welfare regime

**Notes**: All models adjust for sex, age at death, marital status, and proxy relationship. Models conditional on prior disability additionally adjust for disability status at the last observation alive and observation gap. Error bars represent 95% confidence intervals. Data are from the Survey of Health, Ageing and Retirement in Europe.

6. **Robustness checks – Extended descriptive statistics table**

**Table S4.** Descriptive statistics of decedents in SHARE Waves 8 and 9 (*N* = 6,957)

|  | **Welfare regime - Mean (SD) / N (%)** | | | | | | | |
| --- | --- | --- | --- | --- | --- | --- | --- | --- |
| **Variables** | Eastern | | Bismarckian | | Southern | | Nordic | |
| *Disability (last year of life)* |  |  |  |  |  |  |  |  |
| ADL limitations | 2.26 | (2.51) | 3.07 | (2.52) | 2.61 | (2.56) | 2.49 | (2.39) |
| IADL limitations | 3.15 | (2.90) | 3.83 | (2.84) | 3.48 | (2.96) | 3.53 | (2.78) |
| *Disability (last observation alive)* |  |  |  |  |  |  |  |  |
| ADL limitations | 1.04 | (1.79) | 1.21 | (1.92) | 1.22 | (2.01) | 0.88 | (1.66) |
| IADL limitations | 1.73 | (2.32) | 1.93 | (2.41) | 1.93 | (2.48) | 1.44 | (2.13) |
| *Observation gap* | 2.30 | (1.89) | 2.72 | (2.20) | 2.71 | (2.52) | 2.63 | (2.32) |
| *Educational attainment* |  |  |  |  |  |  |  |  |
| Low | 1,642 | (47.7%) | 617 | (43.5%) | 1,127 | (79.6%) | 315 | (46.5%) |
| Medium | 1,407 | (40.8%) | 522 | (36.8%) | 192 | (13.6%) | 207 | (30.6%) |
| High | 396 | (11.5%) | 280 | (19.7%) | 97 | (6.85%) | 155 | (22.9%) |
| *Sex* |  |  |  |  |  |  |  |  |
| Male | 1,768 | (51.3%) | 791 | (55.7%) | 727 | (51.3%) | 360 | (53.2%) |
| Female | 1,677 | (48.7%) | 628 | (44.3%) | 689 | (48.7%) | 317 | (46.8%) |
| *Age at death (years)* | 78.8 | (9.88) | 81.6 | (9.98) | 82.6 | (9.06) | 82.0 | (9.55) |
| *Marital status* |  |  |  |  |  |  |  |  |
| Married | 1,688 | (49.0%) | 795 | (56.0%) | 784 | (55.4%) | 332 | (49.0%) |
| Not married | 1,757 | (51.0%) | 624 | (44.0%) | 632 | (44.6%) | 345 | (51.0%) |
| *Proxy* |  |  |  |  |  |  |  |  |
| Spouse | 1,340 | (38.9%) | 660 | (46.5%) | 477 | (33.7%) | 263 | (38.9%) |
| Other relative | 1,428 | (41.5%) | 580 | (40.9%) | 743 | (52.5%) | 404 | (59.7%) |
| Non-relative | 677 | (19.7%) | 179 | (12.6%) | 196 | (13.8%) | 10 | (1.48%) |

7. **Robustness checks – Collapsing educational groups**

**Table S5.** Random intercept beta-binomial (logit) regressions of ADL and IADL limitations at the end of life with collapsed education groups, SHARE decedents (Waves 8 and 9, *N* = 6,957; 27 countries)

|  | **Model** | | | |
| --- | --- | --- | --- | --- |
| **Predictor** | (1) ADL | (2) ADL | (3) IADL | (4) IADL |
| *Educational attainment* |  |  |  |  |
| Low | ref. | ref. | ref. | ref. |
| Medium/High | 0.81*** (0.74–0.88) | 0.86*** (0.78–0.94) | 0.76*** (0.70–0.83) | 0.85*** (0.78–0.93) |
| *Sex* |  |  |  |  |
| Male | ref. | ref. | ref. | ref. |
| Female | 1.31*** (1.20–1.43) | 1.20*** (1.10–1.32) | 1.29*** (1.19–1.41) | 1.15** (1.05–1.25) |
| *Age at death (years)* | 1.04*** (1.04–1.05) | 1.03*** (1.02–1.03) | 1.05*** (1.04–1.05) | 1.03*** (1.03–1.04) |
| *Marital status* |  |  |  |  |
| Married | ref. | ref. | ref. | ref. |
| Not married | 0.99 (0.88–1.11) | 1.00 (0.89–1.13) | 1.02 (0.92–1.14) | 1.00 (0.89–1.12) |
| *Proxy* |  |  |  |  |
| Spouse | ref. | ref. | ref. | ref. |
| Other relative | 0.83** (0.73–0.94) | 0.81*** (0.71–0.91) | 0.98 (0.87–1.11) | 0.95 (0.84–1.08) |
| Non-relative | 0.53*** (0.45–0.62) | 0.53*** (0.45–0.62) | 0.66*** (0.57–0.76) | 0.68*** (0.59–0.80) |
| *Prior disability* |  |  |  |  |
| No limitations |  | ref. |  | ref. |
| 1 limitation |  | 1.71*** (1.50–1.94) |  | 1.50*** (1.33–1.69) |
| 2 limitations |  | 2.29*** (1.96–2.68) |  | 1.95*** (1.70–2.25) |
| 3 limitations |  | 3.13*** (2.58–3.78) |  | 2.19*** (1.87–2.57) |
| 4 limitations |  | 3.34*** (2.64–4.22) |  | 2.85*** (2.39–3.39) |
| 5 limitations |  | 4.87*** (3.90–6.07) |  | 3.56*** (2.94–4.31) |
| 6 limitations |  | 7.44*** (6.17–8.97) |  | 5.39*** (4.33–6.72) |
| 7 limitations |  |  |  | 8.97*** (7.48–10.8) |
| *Observation gap (years)* |  | 1.06*** (1.04–1.08) |  | 1.07*** (1.05–1.09) |
| *Model parameters* |  |  |  |  |
| Intercept (log-odds) | −3.408*** | −2.991*** | −3.890*** | −3.138*** |
| Dispersion (*φ*) | 0.523 | 0.596 | 0.638 | 0.735 |
| Country SD | 0.339 | 0.306 | 0.324 | 0.287 |
| *Model fit* |  |  |  |  |
| Log-likelihood | −11,036.63 | −10,650.69 | −12,246.60 | −11,803.07 |
| AIC | 22,091.26 | 21,333.38 | 24,511.19 | 23,640.15 |
| BIC | 22,152.89 | 21,442.94 | 24,572.82 | 23,756.55 |

**Notes**: Odds ratios (exponentiated coefficients) with 95% CIs in parentheses. Intercept presented as log-odds. Significance levels: * p<0.05, ** p<0.01, *** p<0.001.

**Table S6.** Random intercept beta-binomial (logit) regressions of ADL limitations at the end of life with collapsed education groups, including welfare regime, SHARE decedents (Waves 8 and 9, *N* = 6,957; 27 countries)

|  | **Model** | | | |
| --- | --- | --- | --- | --- |
| **Predictor** | (1) ADL | (2) ADL | (3) ADL | (4) ADL |
| *Educational attainment* |  |  |  |  |
| Low | ref. | ref. | ref. | ref. |
| Medium/High | 0.81*** (0.74–0.88) | 0.79*** (0.70–0.90) | 0.86*** (0.78–0.94) | 0.84** (0.74–0.95) |
| *Sex* |  |  |  |  |
| Male | ref. | ref. | ref. | ref. |
| Female | 1.31*** (1.20–1.43) | 1.31*** (1.20–1.43) | 1.20*** (1.10–1.32) | 1.20*** (1.10–1.32) |
| *Age at death (years)* | 1.04*** (1.04–1.05) | 1.04*** (1.04–1.05) | 1.03*** (1.02–1.03) | 1.03*** (1.02–1.03) |
| *Marital status* |  |  |  |  |
| Married | ref. | ref. | ref. | ref. |
| Not married | 0.99 (0.88–1.12) | 0.99 (0.89–1.12) | 1.00 (0.89–1.13) | 1.00 (0.89–1.13) |
| *Proxy* |  |  |  |  |
| Spouse | ref. | ref. | ref. | ref. |
| Other relative | 0.83** (0.73–0.94) | 0.82** (0.73–0.93) | 0.81** (0.71–0.92) | 0.80*** (0.71–0.91) |
| Non-relative | 0.53*** (0.45–0.62) | 0.53*** (0.45–0.62) | 0.53*** (0.45–0.63) | 0.53*** (0.45–0.62) |
| *Welfare regime* |  |  |  |  |
| Eastern | ref. | ref. | ref. | ref. |
| Bismarckian | 1.56** (1.16–2.10) | 1.74*** (1.26–2.40) | 1.57*** (1.20–2.04) | 1.73*** (1.29–2.31) |
| Southern | 1.28 (0.93–1.75) | 1.23 (0.89–1.71) | 1.26 (0.95–1.67) | 1.20 (0.90–1.61) |
| Nordic | 1.05 (0.70–1.59) | 0.88 (0.57–1.36) | 1.13 (0.79–1.63) | 0.96 (0.65–1.42) |
| *Education × Welfare regime* |  |  |  |  |
| Low × Eastern |  | ref. |  | ref. |
| Medium/High × Bismarckian |  | 0.82 (0.66–1.02) |  | 0.84 (0.67–1.05) |
| Medium/High × Southern |  | 1.15 (0.89–1.49) |  | 1.19 (0.92–1.55) |
| Medium/High × Nordic |  | 1.40* (1.06–1.85) |  | 1.37* (1.03–1.81) |
| *Prior disability* |  |  |  |  |
| No limitations |  |  | ref. | ref. |
| 1 limitation |  |  | 1.71*** (1.50–1.94) | 1.71*** (1.51–1.94) |
| 2 limitations |  |  | 2.30*** (1.96–2.69) | 2.29*** (1.96–2.68) |
| 3 limitations |  |  | 3.14*** (2.59–3.80) | 3.14*** (2.59–3.80) |
| 4 limitations |  |  | 3.33*** (2.64–4.21) | 3.34*** (2.64–4.22) |
| 5 limitations |  |  | 4.88*** (3.91–6.09) | 4.89*** (3.92–6.10) |
| 6 limitations |  |  | 7.45*** (6.18–8.99) | 7.44*** (6.16–8.97) |
| *Observation gap (years)* |  |  | 1.06*** (1.04–1.08) | 1.06*** (1.04–1.08) |
| *Model parameters* |  |  |  |  |
| Intercept (log-odds) | −3.555*** | −3.562*** | −3.134*** | −3.134*** |
| Dispersion (*φ*) | 0.523 | 0.524 | 0.595 | 0.596 |
| Country SD | 0.284 | 0.286 | 0.243 | 0.244 |
| *Model fit* |  |  |  |  |
| Log-likelihood | −11,032.59 | −11,026.47 | −10,645.90 | −10,640.48 |
| AIC | 22,089.19 | 22,082.94 | 21,329.80 | 21,324.96 |
| BIC | 22,171.36 | 22,185.65 | 21,459.90 | 21,475.61 |
| Likelihood ratio test |  | 12.25** |  | 10.84* |

**Notes**: Odds ratios (exponentiated coefficients) with 95% CIs in parentheses. Intercept presented as log-odds. Significance levels: * p<0.05, ** p<0.01, *** p<0.001. Likelihood ratio test compares the additive models (Models 1 and 3) to the interaction models (Models 2 and 4).

**Table S7.** Random intercept beta-binomial (logit) regressions of IADL limitations at the end of life with collapsed education groups, including welfare regime, SHARE decedents (Waves 8 and 9, *N* = 6,957; 27 countries)

|  | **Model** | | | |
| --- | --- | --- | --- | --- |
| **Predictor** | (1) IADL | (2) IADL | (3) IADL | (4) IADL |
| *Educational attainment* |  |  |  |  |
| Low | ref. | ref. | ref. | ref. |
| Medium/High | 0.76*** (0.70–0.83) | 0.75*** (0.67–0.85) | 0.85*** (0.78–0.93) | 0.84** (0.74–0.94) |
| *Sex* |  |  |  |  |
| Male | ref. | ref. | ref. | ref. |
| Female | 1.29*** (1.19–1.41) | 1.29*** (1.18–1.40) | 1.15** (1.05–1.25) | 1.15** (1.05–1.25) |
| *Age at death (years)* | 1.05*** (1.04–1.05) | 1.05*** (1.04–1.05) | 1.03*** (1.03–1.04) | 1.03*** (1.03–1.04) |
| *Marital status* |  |  |  |  |
| Married | ref. | ref. | ref. | ref. |
| Not married | 1.02 (0.92–1.14) | 1.02 (0.92–1.15) | 1.00 (0.89–1.12) | 1.00 (0.89–1.12) |
| *Proxy* |  |  |  |  |
| Spouse | ref. | ref. | ref. | ref. |
| Other relative | 0.98 (0.87–1.11) | 0.98 (0.87–1.10) | 0.95 (0.85–1.08) | 0.95 (0.84–1.07) |
| Non-relative | 0.66*** (0.57–0.77) | 0.66*** (0.57–0.76) | 0.69*** (0.59–0.80) | 0.69*** (0.59–0.80) |
| *Welfare regime* |  |  |  |  |
| Eastern | ref. | ref. | ref. | ref. |
| Bismarckian | 1.33 (0.97–1.82) | 1.47* (1.05–2.06) | 1.29 (0.97–1.70) | 1.40* (1.03–1.89) |
| Southern | 1.14 (0.82–1.59) | 1.11 (0.79–1.56) | 1.12 (0.83–1.51) | 1.08 (0.79–1.46) |
| Nordic | 1.14 (0.74–1.76) | 0.98 (0.62–1.55) | 1.27 (0.86–1.86) | 1.12 (0.74–1.70) |
| *Education × Welfare regime* |  |  |  |  |
| Low × Eastern |  | ref. |  | ref. |
| Medium/High × Bismarckian |  | 0.83 (0.67–1.03) |  | 0.86 (0.70–1.07) |
| Medium/High × Southern |  | 1.12 (0.87–1.43) |  | 1.15 (0.90–1.48) |
| Medium/High × Nordic |  | 1.31 (1.00–1.72) |  | 1.25 (0.95–1.64) |
| *Prior disability* |  |  |  |  |
| No limitations |  |  | ref. | ref. |
| 1 limitation |  |  | 1.50*** (1.33–1.69) | 1.50*** (1.33–1.69) |
| 2 limitations |  |  | 1.95*** (1.69–2.25) | 1.96*** (1.70–2.25) |
| 3 limitations |  |  | 2.19*** (1.87–2.57) | 2.19*** (1.87–2.57) |
| 4 limitations |  |  | 2.85*** (2.39–3.39) | 2.84*** (2.38–3.38) |
| 5 limitations |  |  | 3.56*** (2.94–4.31) | 3.53*** (2.91–4.28) |
| 6 limitations |  |  | 5.39*** (4.32–6.71) | 5.40*** (4.33–6.72) |
| 7 limitations |  |  | 8.99*** (7.49–10.8) | 8.98*** (7.49–10.8) |
| *Observation gap (years)* |  |  | 1.07*** (1.04–1.09) | 1.06*** (1.04–1.09) |
| *Model parameters* |  |  |  |  |
| Intercept (log-odds) | −3.990*** | −3.997*** | −3.231*** | −3.233*** |
| Dispersion (*φ*) | 0.638 | 0.639 | 0.735 | 0.736 |
| Country SD | 0.304 | 0.304 | 0.265 | 0.265 |
| *Model fit* |  |  |  |  |
| Log-likelihood | −12,245.06 | −12,240.32 | −11,801.34 | −11,797.88 |
| AIC | 24,514.12 | 24,510.63 | 23,642.69 | 23,641.75 |
| BIC | 24,596.29 | 24,613.34 | 23,779.64 | 23,799.25 |
| Likelihood ratio test |  | 9.48* |  | 6.93 |

**Notes**: Odds ratios (exponentiated coefficients) with 95% CIs in parentheses. Intercept presented as log-odds. Significance levels: * p<0.05, ** p<0.01, *** p<0.001. Likelihood ratio test compares the additive models (Models 1 and 3) to the interaction models (Models 2 and 4).

8. **Robustness checks – Contrasts using collapsed education groups**

**Figure S5.** Absolute differences in predicted ADL limitations between medium- to high-educated decedents relative to low-educated decedents, by European welfare regime

**Notes**: All models adjust for sex, age at death, marital status, and proxy relationship. Models conditional on prior disability additionally adjust for disability status at the last observation alive and observation gap. Error bars represent 95% confidence intervals. Data are from the Survey of Health, Ageing and Retirement in Europe.

**Figure S6.** Absolute differences in predicted IADL limitations between medium- to high-educated decedents relative to low-educated decedents, by European welfare regime

**Notes**: All models adjust for sex, age at death, marital status, and proxy relationship. Models conditional on prior disability additionally adjust for disability status at the last observation alive and observation gap. Error bars represent 95% confidence intervals. Data are from the Survey of Health, Ageing and Retirement in Europe.

9. **Robustness checks – Less gendered IADL activities**

**Table S8.** Random intercept beta-binomial (logit) regressions of IADL limitations at the end of life with less gendered activities, SHARE decedents (Waves 8 and 9, *N* = 6,957; 27 countries)

|  | **Model** | |
| --- | --- | --- |
| **Predictor** | (1) Less gendered IADL | (2) Less gendered IADL |
| *Educational attainment* |  |  |
| Low | ref. | ref. |
| Medium | 0.74*** (0.66–0.83) | 0.79*** (0.70–0.88) |
| High | 0.65*** (0.55–0.76) | 0.75*** (0.61–0.84) |
| *Sex* |  |  |
| Male | ref. | ref. |
| Female | 1.19** (1.07–1.32) | 1.12* (1.01–1.25) |
| *Age at death (years)* | 1.05*** (1.04–1.06) | 1.03*** (1.03–1.04) |
| *Marital status* |  |  |
| Married | ref. | ref. |
| Not married | 1.00 (0.87–1.14) | 1.00 (0.87–1.16) |
| *Proxy* |  |  |
| Spouse | ref. | ref. |
| Other relative | 0.93 (0.80–1.08) | 0.93 (0.80–1.08) |
| Non-relative | 0.56*** (0.46–0.67) | 0.60*** (0.49–0.73) |
| *Prior disability* |  |  |
| No limitations |  | ref. |
| 1 limitation |  | 3.00*** (2.55–3.53) |
| 2 limitations |  | 7.76*** (6.50–9.26) |
| *Observation gap (years)* |  | 1.06*** (1.04–1.08) |
| *Model parameters* |  |  |
| Intercept (log-odds) | −4.331*** | −3.609*** |
| Dispersion (*φ*) | 0.404 | 0.463 |
| Country SD | 0.373 | 0.329 |
| *Model fit* |  |  |
| Log-likelihood | −6,114.38 | −5,771.10 |
| AIC | 12,248.75 | 11,568.19 |
| BIC | 12,248.75 | 11,657.21 |

**Notes**: Odds ratios (exponentiated coefficients) with 95% CIs in parentheses. Intercept presented as log-odds. Significance levels: * p<0.05, ** p<0.01, *** p<0.001.

10. **Robustness checks – Interaction models**

**Table S9.** Random intercept beta-binomial (logit) regressions of ADL and IADL limitations at the end of life, including sex × educational attainment interaction, SHARE decedents (Waves 8 and 9, *N* = 6,957; 27 countries)

|  | **Model** | | | |
| --- | --- | --- | --- | --- |
| **Predictor** | (1) ADL | (2) ADL | (3) IADL | (4) IADL |
| *Educational attainment* |  |  |  |  |
| Low | ref. | ref. | ref. | ref. |
| Medium | 0.86* (0.76–0.98) | 0.89 (0.78–1.01) | 0.81*** (0.71–0.91) | 0.87* (0.77–0.99) |
| High | 0.75*** (0.63–0.88) | 0.81* (0.69–0.96) | 0.67*** (0.57–0.79) | 0.79** (0.67–0.92) |
| *Sex* |  |  |  |  |
| Male | ref. | ref. | ref. | ref. |
| Female | 1.33*** (1.19–1.50) | 1.21** (1.07–1.36) | 1.29*** (1.16–1.45) | 1.14* (1.02–1.28) |
| *Sex × Educational attainment* |  |  |  |  |
| Male × Low | ref. | ref. | ref. | ref. |
| Female × Medium | 0.99 (0.83–1.19) | 1.03 (0.85–1.23) | 0.97 (0.82–1.16) | 0.99 (0.83–1.18) |
| Female × High | 0.84 (0.64–1.09) | 0.86 (0.66–1.13) | 1.03 (0.81–1.33) | 1.06 (0.82–1.36) |
| *Age at death (years)* | 1.04*** (1.04–1.05) | 1.03*** (1.02–1.03) | 1.05*** (1.05–1.05) | 1.03*** (1.03–1.04) |
| *Marital status* |  |  |  |  |
| Married | ref. | ref. | ref. | ref. |
| Not married | 0.99 (0.88–1.11) | 1.00 (0.89–1.13) | 1.02 (0.91–1.14) | 1.00 (0.89–1.12) |
| *Proxy* |  |  |  |  |
| Spouse | ref. | ref. | ref. | ref. |
| Other relative | 0.64*** (0.57–0.73) | 0.66*** (0.58–0.75) | 0.67*** (0.60–0.76) | 0.72*** (0.64–0.81) |
| Non-relative | 1.21** (1.07–1.37) | 1.24*** (1.09–1.41) | 1.02 (0.90–1.15) | 1.05 (0.93–1.19) |
| *Prior disability* |  |  |  |  |
| No limitations |  | ref. |  | ref. |
| 1 limitation |  | 1.70*** (1.50–1.93) |  | 1.50*** (1.33–1.69) |
| 2 limitations |  | 2.29*** (1.96–2.68) |  | 1.94*** (1.69–2.24) |
| 3 limitations |  | 3.11*** (2.57–3.77) |  | 2.19*** (1.87–2.56) |
| 4 limitations |  | 3.32*** (2.63–4.20) |  | 2.84*** (2.38–3.38) |
| 5 limitations |  | 4.86*** (3.89–6.06) |  | 3.55*** (2.93–4.30) |
| 6 limitations |  | 7.42*** (6.15–8.94) |  | 5.36*** (4.30–6.68) |
| 7 limitations |  |  |  | 8.96*** (7.47–10.7) |
| *Observation gap (years)* |  | 1.06*** (1.04–1.08) |  | 1.07*** (1.05–1.09) |
| *Model parameters* |  |  |  |  |
| Intercept (log-odds) | −3.625*** | −3.226*** | −3.939*** | −3.206*** |
| Dispersion ($\varphi$) | 0.524 | 0.596 | 0.639 | 0.735 |
| Country SD | 0.342 | 0.308 | 0.325 | 0.288 |
| *Model fit* |  |  |  |  |
| Log-likelihood | −11031.5 | −10647.7 | −12243.6 | −11802.2 |
| AIC | 22087.0 | 21333.4 | 24511.1 | 23644.4 |
| BIC | 22169.1 | 21463.5 | 24593.3 | 23781.4 |
| Likelihood ratio test | 1.79 | 1.49 | 0.26 | 0.88 |

**Notes**: Odds ratios (exponentiated coefficients) with 95% CIs in parentheses. Intercept presented as log-odds. Significance levels: * p<0.05, ** p<0.01, *** p<0.001. Likelihood ratio test compares the models to the corresponding main models of Table S1.

**Table S10.** Random intercept beta-binomial (logit) regressions of ADL limitations at the end of life, including categorical age at death × educational attainment interaction, SHARE decedents (Waves 8 and 9, *N* = 6,957; 27 countries)

|  | **Model** | | | |
| --- | --- | --- | --- | --- |
| **Predictor** | (1) ADL | (2) ADL | (3) ADL | (4) ADL |
| *Educational attainment* |  |  |  |  |
| Low | ref. | ref. | ref. | ref. |
| Medium | 0.84*** (0.76–0.92) | 0.84 (0.71–1.01) | 0.88* (0.80–0.98) | 0.83* (0.70–0.99) |
| High | 0.69*** (0.61–0.79) | 0.65** (0.50–0.84) | 0.77*** (0.67–0.88) | 0.70** (0.54–0.91) |
| *Sex* |  |  |  |  |
| Male | ref. | ref. | ref. | ref. |
| Female | 1.30*** (1.19–1.42) | 1.30*** (1.19–1.43) | 1.20*** (1.09–1.31) | 1.20*** (1.10–1.32) |
| *Age at death (years)* |  |  |  |  |
| <75 | ref. | ref. | ref. | ref. |
| 75–85 | 1.55*** (1.39–1.73) | 1.51*** (1.29–1.77) | 1.38*** (1.24–1.54) | 1.31*** (1.12–1.54) |
| 85> | 2.51*** (2.24–2.82) | 2.52*** (2.16–2.95) | 1.93*** (1.72–2.16) | 1.83*** (1.56–2.15) |
| *Age at death × Education* |  |  |  |  |
| <75 × Low | ref. | ref. | ref. | ref. |
| 75–85 × Medium |  | 1.04 (0.83–1.32) |  | 1.09 (0.86–1.38) |
| 75–85 × High |  | 1.08 (0.78–1.52) |  | 1.10 (0.79–1.55) |
| 85> × Medium |  | 0.93 (0.74–1.18) |  | 1.08 (0.85–1.36) |
| 85> × High |  | 1.11 (0.80–1.54) |  | 1.15 (0.82–1.60) |
| *Marital status* |  |  |  |  |
| Married | ref. | ref. | ref. | ref. |
| Not married | 1.00 (0.89–1.12) | 1.00 (0.89–1.12) | 1.01 (0.90–1.13) | 1.01 (0.90–1.13) |
| *Proxy* |  |  |  |  |
| Spouse | ref. | ref. | ref. | ref. |
| Other relative | 0.64*** (0.56–0.73) | 0.64*** (0.56–0.73) | 0.66*** (0.58–0.75) | 0.66*** (0.58–0.75) |
| Non-relative | 1.20** (1.06–1.36) | 1.20** (1.06–1.36) | 1.23** (1.09–1.40) | 1.23** (1.09–1.40) |
| *Prior disability* |  |  |  |  |
| No limitations |  |  | ref. | ref. |
| 1 limitation |  |  | 1.71*** (1.51–1.95) | 1.71*** (1.51–1.94) |
| 2 limitations |  |  | 2.32*** (1.99–2.72) | 2.32*** (1.99–2.72) |
| 3 limitations |  |  | 3.12*** (2.57–3.78) | 3.12*** (2.57–3.78) |
| 4 limitations |  |  | 3.40*** (2.69–4.29) | 3.40*** (2.69–4.30) |
| 5 limitations |  |  | 4.94*** (3.95–6.16) | 4.94*** (3.96–6.16) |
| 6 limitations |  |  | 7.51*** (6.22–9.05) | 7.52*** (6.23–9.07) |
| *Observation gap (years)* |  |  | 1.06*** (1.04–1.08) | 1.06*** (1.04–1.08) |
| *Model parameters* |  |  |  |  |
| Intercept (log-odds) | −0.829*** | −0.823*** | −1.258*** | −1.219*** |
| Dispersion ($\varphi$) | 0.521 | 0.521 | 0.595 | 0.595 |
| Country SD | 0.345 | 0.345 | 0.310 | 0.310 |
| *Model fit* |  |  |  |  |
| Log-likelihood | −11,047.5 | −11,046.7 | −10,655.9 | −10,655.4 |
| AIC | 22,117.1 | 22,123.4 | 21,347.7 | 21,354.7 |
| BIC | 22,192.4 | 22,226.1 | 21,471.0 | 21,505.4 |
| Likelihood ratio test |  | 1.72 |  | 1.01 |

**Notes**: Odds ratios (exponentiated coefficients) with 95% CIs in parentheses. Intercept presented as log-odds. Significance levels: * p<0.05, ** p<0.01, *** p<0.001. Likelihood ratio test compares the models (Models 1 and 3) to the interaction models (Models 2 and 4).

**Table S11.** Random intercept beta-binomial (logit) regressions of IADL limitations at the end of life, including categorical age at death × educational attainment interaction, SHARE decedents (Waves 8 and 9, *N* = 6,957; 27 countries)

|  | **Model** | | | |
| --- | --- | --- | --- | --- |
| **Predictor** | (1) IADL | (2) IADL | (3) IADL | (4) IADL |
| *Educational attainment* |  |  |  |  |
| Low | ref. | ref. | ref. | ref. |
| Medium | 0.78*** (0.71–0.85) | 0.82* (0.70–0.97) | 0.86** (0.78–0.94) | 0.86 (0.73–1.01) |
| High | 0.67*** (0.59–0.76) | 0.72** (0.57–0.92) | 0.80*** (0.70–0.91) | 0.83 (0.65–1.05) |
| *Sex* |  |  |  |  |
| Male | ref. | ref. | ref. | ref. |
| Female | 1.29*** (1.19–1.41) | 1.29*** (1.18–1.40) | 1.15** (1.05–1.25) | 1.14** (1.05–1.25) |
| *Age at death (years)* |  |  |  |  |
| <75 | ref. | ref. | ref. | ref. |
| 75–85 | 1.76*** (1.59–1.95) | 1.80*** (1.55–2.10) | 1.49*** (1.34–1.65) | 1.49*** (1.28–1.73) |
| 85> | 2.96*** (2.66–3.30) | 3.16*** (2.72–3.67) | 1.99*** (1.78–2.22) | 2.02*** (1.73–2.35) |
| *Age at death × Education* |  |  |  |  |
| <75 × Low |  | ref. |  | ref. |
| 75–85 × Medium |  | 0.97 (0.78–1.20) |  | 1.00 (0.80–1.25) |
| 75–85 × High |  | 0.97 (0.71–1.32) |  | 1.00 (0.73–1.36) |
| 85> × Medium |  | 0.88 (0.70–1.10) |  | 1.00 (0.80–1.25) |
| 85> × High |  | 0.85 (0.62–1.16) |  | 0.90 (0.66–1.23) |
| *Marital status* |  |  |  |  |
| Married | ref. | ref. | ref. | ref. |
| Not married | 1.03 (0.92–1.15) | 1.03 (0.92–1.15) | 1.00 (0.90–1.12) | 1.00 (0.90–1.12) |
| *Proxy* |  |  |  |  |
| Spouse | ref. | ref. | ref. | ref. |
| Other relative | 1.00 (0.89–1.13) | 1.00 (0.89–1.13) | 1.03 (0.92–1.17) | 1.03 (0.92–1.17) |
| Non-relative | 0.67*** (0.59–0.75) | 0.67*** (0.59–0.75) | 0.72*** (0.64–0.81) | 0.72*** (0.64–0.81) |
| *Prior disability* |  |  |  |  |
| No limitations |  |  | ref. | ref. |
| 1 limitation |  |  | 1.50*** (1.33–1.69) | 1.50*** (1.33–1.69) |
| 2 limitations |  |  | 1.97*** (1.71–2.26) | 1.97*** (1.71–2.27) |
| 3 limitations |  |  | 2.22*** (1.89–2.60) | 2.22*** (1.89–2.60) |
| 4 limitations |  |  | 2.91*** (2.44–3.46) | 2.91*** (2.44–3.46) |
| 5 limitations |  |  | 3.64*** (3.00–4.41) | 3.64*** (3.00–4.41) |
| 6 limitations |  |  | 5.55*** (4.46–6.92) | 5.56*** (4.46–6.92) |
| 7 limitations |  |  | 9.23*** (7.70–11.1) | 9.22*** (7.69–11.1) |
| *Observation gap (years)* |  |  | 1.07*** (1.05–1.09) | 1.07*** (1.05–1.09) |
| *Model parameters* |  |  |  |  |
| Intercept (log-odds) | –0.603*** | –0.638*** | –1.088*** | –1.094*** |
| Dispersion ($\varphi$) | 0.633 | 0.633 | 0.732 | 0.732 |
| Country SD | 0.325 | 0.325 | 0.288 | 0.288 |
| *Model fit* |  |  |  |  |
| Log-likelihood | –12273.3 | –12272.3 | –11815.0 | –11814.7 |
| AIC | 24568.7 | 24574.5 | 23668.1 | 23675.4 |
| BIC | 24644.0 | 24677.2 | 23798.2 | 23832.9 |
| Likelihood ratio test |  | 2.17 |  | 0.71 |

**Notes**: Odds ratios (exponentiated coefficients) with 95% CIs in parentheses. Intercept presented as log-odds. Significance levels: * p<0.05, ** p<0.01, *** p<0.001. Likelihood ratio test compares the models (Models 1 and 3) to the interaction models (Models 2 and 4).

11. **Robustness checks – Welfare regime heterogeneity**

**Table S12.** Sensitivity of welfare regime contrast-estimates to the exclusion of individual countries

| **Excluded country** | **Outcome** | **Model** | **Welfare regime** | **Contrast** | **Estimate** | **Full sample estimate** | **Absolute deviation** |
| --- | --- | --- | --- | --- | --- | --- | --- |
| Sweden | IADL | M4 | Nordic | Medium−Low | –0.050 | 0.030 | 0.080 |
| Sweden | IADL | M6 | Nordic | Medium−Low | –0.041 | 0.030 | 0.071 |
| Sweden | IADL | M4 | Nordic | High−Low | –0.115 | –0.046 | 0.070 |
| Sweden | IADL | M6 | Nordic | High−Low | –0.076 | –0.019 | 0.057 |
| Denmark | IADL | M4 | Nordic | Medium−Low | 0.073 | 0.030 | 0.043 |
| Sweden | ADL | M4 | Nordic | High−Low | –0.043 | –0.001 | 0.043 |
| Italy | ADL | M6 | Southern | Medium−Low | 0.065 | 0.025 | 0.040 |
| Denmark | IADL | M6 | Nordic | Medium−Low | 0.070 | 0.030 | 0.040 |
| Denmark | ADL | M4 | Nordic | High−Low | 0.038 | –0.001 | 0.038 |
| Denmark | IADL | M4 | Nordic | High−Low | –0.007 | –0.046 | 0.038 |
| Greece | ADL | M4 | Southern | Medium−Low | –0.032 | 0.006 | 0.038 |
| Italy | ADL | M4 | Southern | Medium−Low | 0.040 | 0.006 | 0.034 |
| Denmark | ADL | M6 | Nordic | High−Low | 0.048 | 0.015 | 0.033 |
| Denmark | IADL | M6 | Nordic | High−Low | 0.011 | –0.019 | 0.030 |
| Greece | ADL | M6 | Southern | Medium−Low | –0.004 | 0.025 | 0.030 |
| Sweden | ADL | M6 | Nordic | High−Low | –0.014 | 0.015 | 0.029 |
| Portugal | IADL | M4 | Southern | Medium−Low | –0.056 | –0.035 | 0.021 |
| Italy | ADL | M6 | Southern | High−Low | –0.031 | –0.051 | 0.021 |
| Italy | ADL | M4 | Southern | High−Low | –0.059 | –0.079 | 0.020 |
| Estonia | IADL | M6 | Eastern | High−Low | –0.037 | –0.055 | 0.019 |
| Cyprus | ADL | M6 | Southern | High−Low | –0.069 | –0.051 | 0.018 |
| Spain | ADL | M4 | Southern | Medium−Low | 0.024 | 0.006 | 0.018 |
| Cyprus | ADL | M4 | Southern | High−Low | –0.097 | –0.079 | 0.018 |
| Portugal | ADL | M4 | Southern | Medium−Low | –0.012 | 0.006 | 0.018 |
| Sweden | ADL | M4 | Nordic | Medium−Low | 0.026 | 0.043 | 0.017 |
| Austria | IADL | M4 | Bismarckian | High−Low | –0.144 | –0.126 | 0.017 |
| Estonia | ADL | M4 | Eastern | High−Low | –0.076 | –0.093 | 0.017 |
| Portugal | ADL | M6 | Southern | Medium−Low | 0.009 | 0.025 | 0.016 |
| Estonia | ADL | M6 | Eastern | High−Low | –0.055 | –0.070 | 0.015 |
| Portugal | IADL | M6 | Southern | Medium−Low | –0.022 | –0.007 | 0.015 |
| Spain | IADL | M4 | Southern | Medium−Low | –0.020 | –0.035 | 0.015 |
| Italy | IADL | M4 | Southern | High−Low | –0.039 | –0.053 | 0.014 |
| Austria | IADL | M6 | Bismarckian | High−Low | –0.088 | –0.073 | 0.014 |
| Spain | ADL | M6 | Southern | Medium−Low | 0.039 | 0.025 | 0.014 |
| Sweden | ADL | M6 | Nordic | Medium−Low | 0.025 | 0.039 | 0.014 |
| Greece | IADL | M6 | Southern | Medium−Low | 0.007 | –0.007 | 0.014 |
| Spain | IADL | M6 | Southern | Medium−Low | 0.007 | –0.007 | 0.014 |
| Estonia | IADL | M4 | Eastern | High−Low | –0.080 | –0.094 | 0.014 |
| Spain | ADL | M6 | Southern | High−Low | –0.065 | –0.051 | 0.014 |
| Belgium | IADL | M4 | Bismarckian | High−Low | –0.113 | –0.126 | 0.014 |

**Notes**: Table reports the 40 country exclusions associated with the largest absolute deviations from the full-sample estimates in a country-exclusion sensitivity analysis. Models were re-estimated sequentially, excluding one country at a time, and welfare regime contrasts were compared with the corresponding full-sample estimates (absolute predicted ADL and IADL differences). Influence is measured as the absolute difference between the full-sample and re-estimated contrast. Larger values indicate greater sensitivity of the estimated education-by-welfare regime contrasts to the exclusion of a given country. Results are shown for ADL and IADL outcomes and for specifications with and without adjustment for prior disability (M4 and M6; corresponding to Models 2 and 4 in Tables S2 and S3 for ADL and IADL, respectively).

12. **Predicted counts**

**Figure S7.** Predicted ADL and IADL limitations by education groups, global model (*N* = 6,957)

**Notes**: All models adjust for sex, age at death, marital status, and proxy relationship. Error bars represent 95% confidence intervals. Data are from the Survey of Health, Ageing and Retirement in Europe.

**Figure S8.** Predicted ADL and IADL limitations by education groups in Eastern European welfare regimes (*n* = 3,445)

**Notes**: All models adjust for sex, age at death, marital status, and proxy relationship. Error bars represent 95% confidence intervals. Data are from the Survey of Health, Ageing and Retirement in Europe.

**Figure S9.** Predicted ADL and IADL limitations by education groups in Bismarckian welfare regimes (*n* = 1,419)

**Notes**: All models adjust for sex, age at death, marital status, and proxy relationship. Error bars represent 95% confidence intervals. Data are from the Survey of Health, Ageing and Retirement in Europe.

**Figure S10.** Predicted ADL and IADL limitations by education groups in Southern European welfare regimes (*n* = 1,416)

**Notes**: All models adjust for sex, age at death, marital status, and proxy relationship. Error bars represent 95% confidence intervals. Data are from the Survey of Health, Ageing and Retirement in Europe.

**Figure S11.** Predicted ADL and IADL limitations by education groups in Nordic welfare regimes (*n* = 677)

**Notes**: All models adjust for sex, age at death, marital status, and proxy relationship. Error bars represent 95% confidence intervals. Data are from the Survey of Health, Ageing and Retirement in Europe.

13. **Random intercepts of main and interaction models**

**Table S13.** Country-specific random intercepts from beta-binomial (logit) regressions predicting ADL limitations (Models S1–S2 mapping)

| **Country** | **(1)** | **(2)** | **(3)** | **(4)** | **(5)** | **(6)** |  |
| --- | --- | --- | --- | --- | --- | --- | --- |
| Austria | 0.418 | 0.325 | 0.159 | 0.187 | 0.077 | 0.102 |  |
| Germany | 0.345 | 0.276 | 0.095 | 0.149 | 0.039 | 0.090 |  |
| Sweden | –0.213 | –0.110 | –0.102 | –0.068 | –0.058 | –0.030 |  |
| Netherlands | 0.019 | 0.077 | –0.212 | –0.231 | –0.137 | –0.156 |  |
| Spain | 0.022 | –0.088 | –0.038 | –0.020 | –0.121 | –0.101 |  |
| Italy | –0.109 | –0.116 | –0.168 | –0.173 | –0.152 | –0.158 |  |
| France | 0.273 | 0.267 | 0.028 | –0.016 | 0.033 | –0.006 |  |
| Denmark | –0.106 | –0.091 | –0.001 | –0.043 | –0.041 | –0.076 |  |
| Greece | –0.621 | –0.491 | –0.665 | –0.668 | –0.507 | –0.511 |  |
| Switzerland | –0.064 | 0.048 | –0.279 | –0.267 | –0.154 | –0.139 |  |
| Belgium | 0.263 | 0.208 | 0.008 | –0.019 | –0.034 | –0.062 |  |
| Czech Republic | –0.087 | –0.224 | –0.269 | –0.049 | –0.048 | –0.088 |  |
| Poland | –0.161 | –0.217 | 0.012 | 0.009 | –0.039 | –0.043 |  |
| Luxembourg | 0.397 | 0.366 | 0.200 | 0.197 | 0.175 | 0.172 |  |
| Hungary | –0.514 | –0.471 | –0.334 | –0.331 | –0.279 | –0.275 |  |
| Portugal | 0.668 | 0.567 | 0.571 | 0.579 | 0.473 | 0.484 |  |
| Slovenia | 0.240 | 0.251 | 0.403 | 0.400 | 0.409 | 0.406 |  |
| Estonia | –0.166 | –0.178 | 0.010 | 0.012 | 0.003 | 0.006 |  |
| Croatia | –0.272 | –0.259 | –0.101 | –0.103 | –0.086 | –0.088 |  |
| Lithuania | 0.121 | 0.024 | 0.260 | 0.267 | 0.159 | 0.167 |  |
| Bulgaria | –0.449 | –0.451 | –0.276 | –0.279 | –0.268 | –0.272 |  |
| Cyprus | 0.534 | 0.465 | 0.434 | 0.439 | 0.361 | 0.367 |  |
| Finland | 0.056 | 0.101 | 0.106 | 0.114 | 0.100 | 0.108 |  |
| Latvia | –0.416 | –0.345 | –0.247 | –0.245 | –0.175 | –0.173 |  |
| Malta | –0.104 | –0.035 | –0.133 | –0.156 | –0.053 | –0.079 |  |
| Romania | 0.072 | 0.021 | 0.224 | 0.219 | 0.169 | 0.163 |  |
| Slovakia | 0.009 | 0.146 | 0.111 | 0.112 | 0.207 | 0.209 |  |

**Notes**: Model numbers correspond to those reported in Supplementary Tables S1 and S2: Models 1–2 correspond to Supplementary Table S1 (Models 1–2), while Models 3–6 correspond to Supplementary Table S2 (Models 1–4). Data are from the Survey of Health, Ageing and Retirement in Europe.

**Table S14.** Country-specific random intercepts from beta-binomial (logit) regressions predicting IADL limitations

| **Country** | **(1)** | **(2)** | **(3)** | **(4)** | **(5)** | **(6)** |  |
| --- | --- | --- | --- | --- | --- | --- | --- |
| Austria | 0.362 | 0.225 | 0.200 | 0.224 | 0.097 | 0.117 |  |
| Germany | 0.195 | 0.115 | 0.041 | 0.089 | –0.006 | 0.035 |  |
| Sweden | –0.143 | 0.004 | –0.166 | –0.137 | –0.111 | –0.091 |  |
| Netherlands | –0.170 | –0.139 | –0.315 | –0.334 | –0.248 | –0.266 |  |
| Spain | –0.063 | –0.169 | –0.075 | –0.069 | –0.161 | –0.151 |  |
| Italy | –0.113 | –0.099 | –0.126 | –0.122 | –0.094 | –0.090 |  |
| France | 0.259 | 0.219 | 0.106 | 0.068 | 0.099 | 0.067 |  |
| Denmark | 0.054 | 0.087 | 0.030 | 0.005 | –0.026 | –0.046 |  |
| Greece | –0.537 | –0.423 | –0.545 | –0.547 | –0.412 | –0.414 |  |
| Switzerland | –0.183 | –0.039 | –0.320 | –0.308 | –0.145 | –0.132 |  |
| Belgium | 0.278 | 0.237 | 0.117 | 0.096 | 0.109 | 0.089 |  |
| Czech Republic | –0.145 | –0.193 | –0.030 | –0.030 | –0.082 | –0.082 |  |
| Poland | –0.016 | –0.063 | 0.099 | 0.098 | 0.046 | 0.044 |  |
| Luxembourg | 0.281 | 0.175 | 0.169 | 0.164 | 0.093 | 0.089 |  |
| Hungary | –0.494 | –0.496 | –0.377 | –0.376 | –0.382 | –0.379 |  |
| Portugal | 0.636 | 0.568 | 0.608 | 0.607 | 0.550 | 0.549 |  |
| Slovenia | 0.485 | 0.417 | 0.594 | 0.592 | 0.518 | 0.515 |  |
| Estonia | –0.079 | –0.112 | 0.039 | 0.039 | 0.000 | 0.001 |  |
| Croatia | –0.299 | –0.286 | –0.186 | –0.187 | –0.179 | –0.180 |  |
| Lithuania | 0.174 | 0.191 | 0.272 | 0.275 | 0.278 | 0.281 |  |
| Bulgaria | –0.469 | –0.432 | –0.357 | –0.358 | –0.326 | –0.327 |  |
| Cyprus | 0.409 | 0.311 | 0.382 | 0.379 | 0.293 | 0.287 |  |
| Finland | 0.160 | 0.219 | 0.136 | 0.132 | 0.138 | 0.137 |  |
| Latvia | –0.389 | –0.310 | –0.282 | –0.280 | –0.211 | –0.208 |  |
| Malta | –0.249 | –0.191 | –0.247 | –0.250 | –0.176 | –0.182 |  |
| Romania | 0.024 | 0.040 | 0.129 | 0.127 | 0.136 | 0.133 |  |
| Slovakia | 0.031 | 0.152 | 0.107 | 0.108 | 0.209 | 0.209 |  |

**Notes**: Model numbers correspond to those reported in Supplementary Tables S1 and S3: Models 3–4 correspond to Supplementary Table S1 (Models 3–4), while Models 3–6 correspond to Supplementary Table S3 (Models 1–4). Data are from the Survey of Health, Ageing and Retirement in Europe.
